# Mammalian Ssu72 phosphatase preferentially considers tissue-specific actively transcribed gene expression by regulating RNA Pol II transcription

**DOI:** 10.7150/thno.62274

**Published:** 2022-01-01

**Authors:** Hyun-Soo Kim, Yoon Jeon, Yoon Ok Jang, Ho Lee, Yong Shin, Chang-Woo Lee

**Affiliations:** 1 Department of Molecular Cell Biology, Sungkyunkwan University School of Medicine, Suwon 16419, South Korea.; 2 Graduate School of Cancer Science and Policy, Research Institute, National Cancer Center, Goyang 10408, South Korea.; 3 Department of Biotechnology, College of Life Science and Biotechnology, Yonsei University, Seoul 03722, South Korea.

**Keywords:** Ssu72 phosphatase, RNA polymerase II, CTD phosphorylation, Transcription, Tissue-selective gene expression, P-TEFb

## Abstract

Reversible phosphorylation of the C-terminal domain (CTD) of RNA polymerase II (Pol II) is essential for gene expression control. How altering the phosphorylation of the CTD contributes to gene expression in mammalian systems remains poorly understood.

**Methods:** Primary mouse embryonic fibroblasts, hepatocytes, and embryonic stem cells were isolated from conditional *Ssu72*^f/f^ mice. To knockout the mouse *Ssu72* gene, we infected the cells with adenoviruses of incorporated luciferase and Cre recombinase, respectively. RNA sequencing, ChIP sequencing, ChIP assay, immunoblot analyses, qRT-PCR assay, and immunostaining were performed to gain insights into the functional mechanisms of Ssu72 loss in Pol II dynamics.

**Results:** Using primary cells isolated from Ssu72 conditional knockout and transgenic mice, we found that mammalian Ssu72-mediated transcriptional elongation rather than polyadenylation or RNA processing contributed to the transcriptional regulation of various genes. Depletion of Ssu72 resulted in aberrant Pol II pausing and elongation defects. Reduced transcriptional elongation efficiency tended to preferentially affect expression levels of actively transcribed genes in a tissue-specific manner. Furthermore, Ssu72 CTD phosphatase seemed to regulate the phosphorylation levels of CTD Ser2 and Thr4 through accurate modulation of P-TEFb activity and recruitment.

**Conclusions:** Our findings demonstrate that mammalian Ssu72 contributes to the transcription of tissue-specific actively transcribed gene expression by regulating reciprocal phosphorylation of Pol II CTD.

## Introduction

Transcription begins with the assembly of a pre-initiation complex at individual gene promoters. This is followed by the initiation of messenger RNA (mRNA) synthesis and the beginning of the elongation process. For all coding genes and several non-coding RNAs [ncRNAs; e.g., small nuclear RNAs (snRNAs) and small nucleolar RNAs (snoRNAs)], recruitment of RNA polymerase II (Pol II) to a promoter is an essential step in transcriptional activation. An interesting and distinct feature of Pol II is the presence of C-terminal domain (CTD) repeats (Tyr1-Ser2-Pro3-Thr4-Ser5-Pro6-Ser7) on its largest subunit (Rpb1), known to play an important role in coordinating accurate transcription, mRNA processing, and histone modification.

The CTD itself is also subjected to extensive post-translational modification during the transcriptional process. The phosphorylation state of Pol II CTD is particularly critical for regulating the recruitment of appropriate transcription-associated factors. When transcription is initiated, Ser5 in the CTD is rapidly phosphorylated by TFIIH-associated kinase Cdk7 (Kin28 in yeast). Levels of phosphorylated Ser5 (pSer5) are decreased during transition from initiation to elongation, concomitant with an increase in the level of pSer2 1. Several phosphatases have been implicated in regulating pSer5 levels. For example, Fcp1 (TFIIF-associated CTD phosphatase 1) is capable of dephosphorylating both pSer2 and pSer5, although it appears to prefer pSer2 2,3. RNA polymerase II-associated protein 2, RPAP2 (Rtr1 in yeast), is another potential pSer5 phosphatase with activity against pSer5, pSer7, and pTyr1 in its CTD, although it does not have phosphatase-active sites 4-6. In recent studies, PP1-PNUTs has suggested as a CTD pSer5 phosphatase 7-9 PNUTS has been shown to bind Pol II and regulate transcription by dephosphorylation of Spt5 and pSer5 of Pol II CTD 9. Integrator-serine/threonine protein phosphatase type 2A (Integrator-PP2A) complex is also identified as a CTD phosphatase, which causes selective dephosphorylation of Spt5 on Ser666 and Pol II CTD on both pSer5 and Ser7 9,10.

Ssu72 has been identified as a Pol II CTD phosphatase with a preference for pSer5 and pSer7. Depletion of Ssu72 in yeast has led to increased levels of both pSer5 and pSer7 before transcriptional termination 11,12. In yeast, Ssu72 can bind to the 3' ends of genes and promote polyadenylation-coupled and polyadenylation-independent termination by interacting with either the cleavage and polyadenylation factor CPF/CPSF or the Nrd1-Nab3-Sen1 termination complex 13,14. At the 3' ends of genes, the phosphatase activity of Ssu72 is stimulated by direct interaction with symplekin (Pta1 in yeast), which is required for polyadenylation and 3'-end cleavage of pre-mRNAs 15,16. However, an extensive targeted proteomic analysis revealed that mammalian Ssu72 is not associated with polyadenylation factors 17.

More recently, the genome-wide occupancy pattern of mammalian Ssu72 in human embryonic stem (ES) cells was found to be opposite to that seen in yeast. The highest peaks are detected in the promoter regions of genes, while small peaks are detected in the termination regions 18. Moreover, Ssu72 may mediate the transition of transcriptional initiation/elongation through dephosphorylation of pSer5 in the proximal promoter 19. Therefore, given the strong link between Ssu72 and the initiation-elongation transition, we posited that Ssu72 might also be required for elongation and accurate gene expression in mammalian cells and tissues. However, the pivotal role of Ssu72 phosphatase in higher eukaryotes remains controversial.

In yeast, Ssu72 has been implicated in cellular viability and efficient transcription of snoRNAs and specific mRNAs but has not been implicated in global gene expression 12,20. It is also reported that mammalian Ssu72 is not significantly involved in cell growth and proliferation of COS7 cells derived from monkey kidneys 21, whereas it is essential for cellular proliferation in cultured chicken B-cells 22. We have demonstrated that human Ssu72 functions as a cohesin-binding phosphatase that regulates chromosomal integrity in human cells 23,24. Due to potential redundancy and conflicts with other phosphatases, it remains unclear whether Ssu72 is indeed involved in the control of cellular viability and specificity by regulating transcriptional initiation and elongation processes in mammals.

Therefore, the objective of this study was to use primary cell lines isolated from Ssu72 conditional knockout (KO) and transgenic mice to examine the specific role of mammalian Ssu72 in regulating RNA Pol II processing during transcription. Loss of mammalian Ssu72 was found to be related to defective transcriptional elongation rather than to failure of transcriptional termination or polyadenylation. In addition, reduced transcriptional elongation seemed to preferentially affect distinct patterns of gene expression of actively transcribed genes in a tissue-specific manner. Furthermore, depletion of Ssu72 resulted in defects of Pol II elongation by reducing CTD Ser2 and Thr4 phosphorylation, which induced Pol II accumulation at the proximal promoter region by increasing CTD Ser5 and Ser7 phosphorylation of actively transcribed genes in a tissue-specific manner. Our results revealed a fundamental role of mammalian Ssu72 in regulating RNA Pol II-mediated transcriptional plasticity for cell-type-specific homeostatic maintenance.

## Results

### Mammalian Ssu72 phosphatase predominantly regulates the expression of actively transcribed gene subsets with cell-type-specificity

To investigate the *in vivo* function of Ssu72 in the transcription and development of mammals, we targeted murine *Ssu72* gene by homologous recombination in mouse embryonic stem cells (Figure S1A-D). The wild type (WT) *Ssu72*^+/+^ and heterozygous *Ssu72*^+/∆^ mice were apparently normal, healthy, and fertile. No developmental abnormalities were detected during 12-month or longer observation period. Although *Ssu72*^∆/∆^ embryos developed to the blastocyst stage, they died at peri-implantation stage (Figure S1E-H), implying that loss of *Ssu72* alleles could lead to embryonic lethality via developmental defects.

We next examined levels of Ssu72 expression during embryogenesis. The levels of Ssu72 were found to be higher during pluripotent stem cell and early embryogenesis than during late embryogenesis (Figure S1I). Similarly, the expression of Ssu72 in ES cells was higher than in differentiated mouse embryonic fibroblasts (MEFs) and hepatocytes, implying that high expression level of Ssu72 in cells might be an important factor for the maintenance of stemness. Inversely, efficient reduction of Ssu72 expression was also required for proper differentiation (Figure S1J).

TUNEL assay showed that *Ssu72*^∆/∆^ embryos exhibited increased apoptotic cell numbers whereas *Ssu72*^+/+^ embryos exhibited little or no apoptotic cells in inner cell mass (Figure S1K-L). We also observed impairment of cell proliferation and DNA replication in *Ssu72*^∆/∆^ embryos based on cell number counting and BrdU incorporation assays (Figure S1M-N), indicating that failure of Ssu72 could result in defects of ES cell maintenance and differentiation. Therefore, we generated *Ssu72*^f/f^ (floxed) mice by flanking the *Ssu72* exon 1 with* loxP* sites, allowing Cre-mediated exon 1 deletion (Figure S1A).

To investigate how mammalian Ssu72 regulated gene expression, we performed RNA sequencing (RNA-Seq) analyses of Ssu72 in three different cell types depending on their proliferation capabilities: ES cells (highly proliferating), MEFs (moderate proliferating), and hepatocytes (almost quiescent). The Ssu72 deletion was induced by infection with a Cre recombinase adenovirus (Ad-Cre) or by intercrossing with albumin-Cre (Alb-Cre; a liver-specific Cre) mice (Figure S2A-C). Infection of ES^f/f^, MEF^f/f^, and hepatocyte^f/f^ with Ad-Cre resulted in an efficient reduction of Ssu72 (Figure S2A-C).

**Figure 1:**
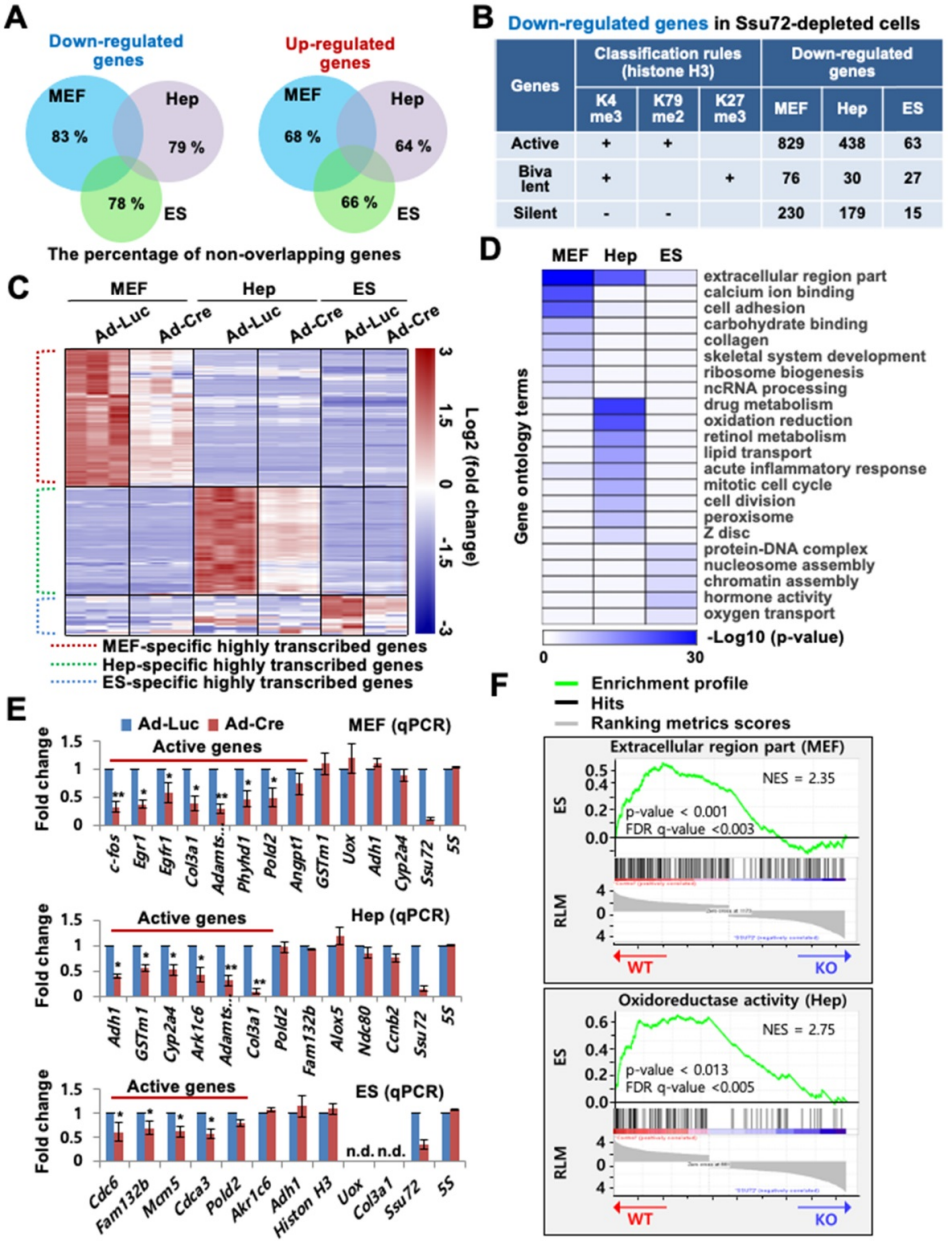
** Mammalian Ssu72 phosphatase predominantly regulates expression of transcriptionally active gene subsets with cell type-specificity. (A)** Venn diagram representing non-overlapping percentages of down- and up-regulated genes in Ssu72-depleted MEFs, hepatocytes, and ES cells. **(B)** Down-regulated genes in MEFs, hepatocytes, and ES cells from RNA-Seq data are classified into transcriptionally active, bivalent, and silent groups based on the presence of co-occupancy of H3K4me3 within ± 2 kb of the transcription start site (TSS) (MEF: GSM307608; Hepatocyte: GSM769014; ES cell: GSM307618), H3K79me2 within the first 5 kb of the gene body (MEF: GSM555115; Hepatocyte: GSM1000152; ES cell: GSM1526289), and H3K27me3 within ± 5 kb of the TSS (MEF: GSM307609; Hepatocyte: GSM1000150; ES cell: GSM307619). “+” denotes the marked histone modification, and “-” represents the unmarked histone modification. **(C)** Heat maps of downregulated genes between control (Ad-Luc infected) and Ssu72-depleted (Ad-Cre infected) MEFs, hepatocytes, and ES cells. Dotted red, green, and blue lines represent specific subsets of transcriptionally active genes in MEFs, hepatocytes, and ES cells, respectively. The color bar represents gradient of log2-fold changes for each cell type. **(D)** Heatmap of gene function's enrichment in various gene ontology categories based on Database for Annotation, Visualization and Integrated Discovery (DAVID). Results are displayed as a sorted heatmap depicting gradient p-value of preferentially altered genes annotated to particular functions. **(E)** qPCR analyses of expression of various genes in control and Ssu72-depleted MEFs, hepatocytes, and ES cells. Red lines represent transcriptionally active genes. Data are representatives of three independent experiments. Error bars indicate standard deviation (SD). 'n.d.' denotes 'not detected'. **(F)** Gene set enrichment analysis (GSEA) enrichment plot of down-regulated gene sets in Ssu72-depleted MEFs and hepatocytes versus related gene sets (MEF: Extracellular region part; Hep: Oxidoreductase activity). The green curve shows the enrichment score, which reflects the degree to which each gene was enriched (black vertical lines).

Depletion of Ssu72 resulted in the up- or down-regulation of large subsets of genes in the three different cell types (Figure 1A-C, Figure S2D, and Table S1). Depletion of Ssu72 resulted in up- and down-regulation [more than 1.5-fold, *p* < 0.05; false discovery rate (FDR) <0.1)] of 902 and 1,135 genes in MEFs, 646 and 654 genes in hepatocytes, and 302 and 125 genes in ES cells, respectively (Table S1). Strikingly, the overall categories of transcripts up- or down-regulated in MEFs, hepatocytes, and ES cells did not significantly overlap (Figure 1A). The percentage of non-overlapping genes in the pool of down-regulated genes was 83% in MEFs, 79% in hepatocytes, and 78% in ES cells; and the percentage of non-overlapping genes in the pool of up-regulated genes was 68% in *Ssu72*-deficient MEFs, 64% in *Ssu72*-deficient hepatocytes, and 66% in Ssu72-deficient ES cells (Figure 1A), indicating that Ssu72 regulated different subsets of genes depending on cell type.

To examine whether Ssu72 regulated gene expression of certain groups, we classified genes altered by Ssu72 depletion to active, bivalent, and silent groups based on co-occupancy with H3K4me3, H3K79me2, and H3K27me3 of previously published RNA-Seq data, respectively (Figure 1B). The percentage of actively transcribed genes in the pool of downregulated genes was 73.0% in Ssu72-deficient MEFs, 67.6% in Ssu72-deficient hepatocytes, and 60.0% in Ssu72-deficient ES cells, while the percentage of silent genes was 20% in MEFs, 27.6% in hepatocytes, and 14% in ES cells (Figure 1B and Table S2). By contrast, we did not find any classification in the pool of upregulated genes.

As expected, our heatmap analyses showed that the down-regulated gene subsets had a substantial population of highly transcribed genes (Figure 1C and Figure S2D), indicating that mammalian Ssu72 might predominantly regulate the expression of actively transcribed genes in a cell-type-specific manner. Gene Ontology (GO) term enrichment analysis also revealed that cellular functionalities of genes altered by Ssu72 depletion were remarkably diverse and dependent on cell type (Figure 1D). Furthermore, based on our RNA-Seq and gene set enrichment analyses (GSEA), we consistently found that the down-regulated gene subsets in Ssu72-depleted MEFs, hepatocytes, and ES cells were significantly correlated with the reduction of the gene sets of GO term analyses in each cell (MEFs: extracellular region part and cell adhesion; hepatocytes: drug metabolism and oxidation-reduction; ES cells: protein-DNA complex and chromatin assembly) (Figure 1D-F, Figure S2E, Table S3, and Table S4).

Additionally, quantitative reverse transcription PCR (qRT-PCR) analysis confirmed that Ssu72 had a tendency to transcriptionally regulate active genes in a cell-type-specific manner, including genes such as *c-fos*, *Egr1*, and *Pold2* in MEFs; *Adh1*, *Gstm1*, and *Ark1c6* in hepatocytes; and *Cdc6*, *Fam132b*, and *Cdca3* in ES cells. These genes were downregulated in the Ssu72-depleted MEFs, hepatocytes, and ES cells (Figure 1E). To further verify that Ssu72 could regulate the expression of specific actively transcribed genes, we generated transgenic mice and MEF cells induced to express HA-tagged Ssu72 (HA-Ssu72) fusion protein at levels similar to endogenous Ssu72 (Figure S2F). Interestingly, immunoblotting and qRT-PCR analyses also revealed that ectopic HA-Ssu72 protein significantly increased the expressed of highly transcribed genes such as *c-fos* and *Egr1* in the condition where the expression of endogenous Ssu72 was inhibited (Figure S2G). These results support the hypothesis that mammalian Ssu72 can predominantly regulate the expression of actively transcribed genes with cell-type specificity.

### Ssu72 is mainly associated with the promoter region of actively transcribed genes in a cell-type-specific manner

**Figure 2:**
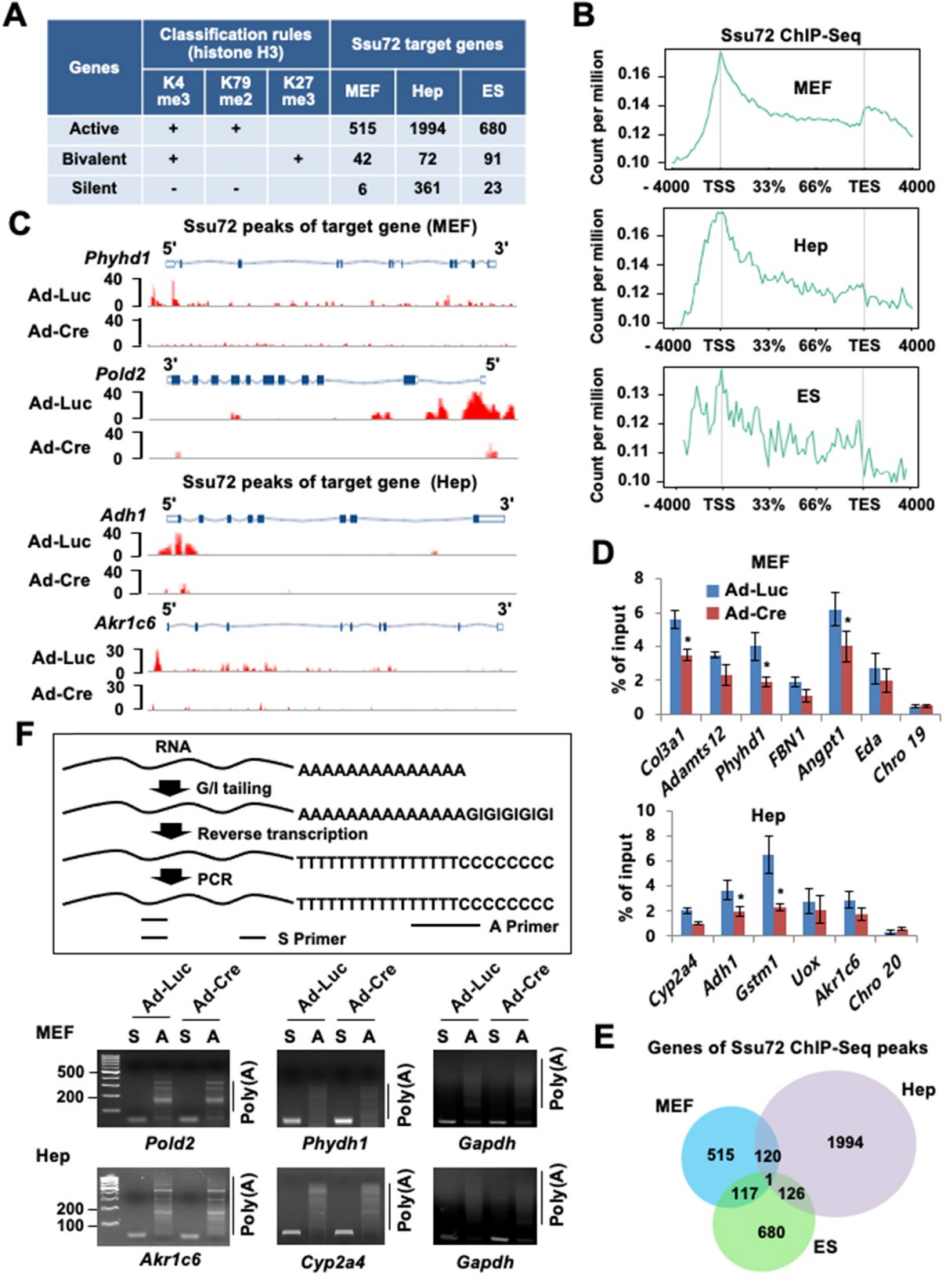
** Ssu72 is mainly localized to the promoter of actively transcribed genes with cell-type specificity. (A)** Ssu72-bound genes based on ChIP-Seq data in MEFs, hepatocytes, and ES cells are classified into transcriptionally active, bivalent, and silent groups based on the presence of co-occupancy of various histone modifications. “+” denotes the marked histone modification, and “-” represents the unmarked histone modification. **(B)** Genome-wide occupancies of high-confident Ssu72 along the transcription unit in MEFs, hepatocytes, and ES cells. Gene expression signals (*y*-axis) are presented according to ChIP-Seq reads per million values. Gene body length (*x*-axis) is displayed as a percentage from the transcription start site (TSS) to the transcription end site (TES). **(C)** Genome browser view of Ssu72 ChIP-Seq signals against* Phyhd1* and *Pold2* genes (Ssu72-bound and target genes, MEFs) and Adh1 and Akr1c6 genes (Ssu72-bound and target genes, hepatocytes) in control and Ssu72-depleted MEFs and hepatocytes. A schematic presentation of mouse Phyhd1, Pold2, Adh1, and Akr1c6 genes is shown in the top panel. **(D)** ChIP and qPCR analyses of core promoter regions within Ssu72-bound genes in control and Ssu72-depleted MEFs and hepatocytes. Random genomic regions of chromosomes 19 and 20 (Chro 19 and Chro 20) are used as negative controls. Data are presented as relative percentage of input recovery (mean value ± SD of three technical replicates).** (E)** Venn diagram presenting overlap of Ssu72-bound genes against high-confidence Ssu72 ChIP-Seq peaks in MEFs, hepatocytes, and ES cells. **(F)** Comparison of mouse poly(A) tail-lengths in MEFs and hepatocytes. Results of Poly(A)-length analyses are displayed in the upper panel. S: gene-specific PCR primer; A: Poly(A) tail PCR primer.

To characterize the relationship between chromatin accessibility of Ssu72 and gene regulation in the three different cell types, we monitored the genome-wide occupancy of Ssu72 in control and Ssu72-deficient MEFs, hepatocytes, and ES cells through chromatin immunoprecipitation (ChIP) coupled with high-throughput sequencing (ChIP-Seq) (Figure 2). In our initial assessment, we specified five distinct genomic regions (intergenic, promoters, exon, intron, and 3'-end) associated with transcriptional regulation in each cell type (Figure S3A). Interestingly, unlike yeast, the occupancies of mammalian Ssu72 in all three cell types were predominantly increased in the promoter regions (Figure S3A). In addition, the heatmap and ChIP-Seq signals of Ssu72 peaks in MEFs also showed a strong co-occupancy with the genomic distribution of Pol II (Rpb1) and Pol II pSer5, which are enriched at the proximal promoter region, but not with those of Pol II pSer2 (Figure S3B-C). To further characterize the distribution of enriched Ssu72 signals within genomic regions, we selected high-confidence Ssu72 ChIP-Seq peaks (4,989 in MEFs, 11,237 in hepatocytes, and 4,020 in ES cells) in the total peaks of each cell type. As a result, we observed 515, 1,994, and 680 Ssu72-bound genes in the MEFs, hepatocytes, and ES cells, respectively (Figure 2A-B, Figure S3A, and Table S5). Interestingly, the metaplot analyses showed that the mammalian Ssu72 ChIP-Seq signals (high-confidence peaks) in all three cell types were increased in the promoter regions (Figure 2B). These results were illustrated using the genome browser view of Ssu72 ChIP-Seq signals against Ssu72-bound genes, such as *Phyhd1* and *Pold2* in MEFs, and *Adh1* and *Akr1c6* in hepatocytes (Figure 2C).

To further validate the recruitment of Ssu72 to the promoter regions, ChIP analysis coupled with qPCR (ChIP-qPCR) was performed for the selected Ssu72-bound genes in the MEFs and hepatocytes (Figure 2D). Ssu72 was found to be markedly enriched in all genomic promoter regions tested, whereas no enrichment was observed in random genomic regions used as negative controls. Importantly, our gene classification analysis revealed that most Ssu72-bound transcripts had H3K4me3- and H3K79me2-modified nucleosomes, which reflected the transcriptionally active genes (Figure 2A). For instance, the percentage of Ssu72-bound genes actively transcribed was 91.5% in MEFs, 82.2% in hepatocytes, and 85.6% in ES cells, while the percentage of silent genes was 1.1% in MEFs, 14.8% in hepatocytes, and 2.9% in ES cells, indicating that the majority of Ssu72-bound transcripts in each cell type were actively transcribed. Additionally, Ssu72-bound genes in MEFs, hepatocytes, and ES cells did not significantly overlap, implying that mammalian Ssu72 can predominantly regulate the expression of actively transcribed genes in a cell-type-dependent manner (Figure 2E). We also performed total Pol II-normalized Ssu72 occupancy analyses in MEFs and hepatocytes. When normalized to Pol II, the occupancy profiles of Ssu72 clearly showed moderate enrichment of Ssu72 in promoter regions compared to the meta-analysis of Ssu72 (Figure S3D). However, we did not observe remarkable changes in the Ssu72 distribution.

Next, to further assess the characteristics of the Ssu72-bound genes by ChIP-Seq data, we integrated the ChIP-Seq results with the genes differentially expressed by Ssu72 depletion (RNA-Seq data; >1.5-fold, FDR < 0.05, *p* < 0.1). Interestingly, a large percentage of Ssu72-bound genes (36.7%) overlapped the genes down-regulated by Ssu72 depletion in MEFs, whereas a small percentage of Ssu72-bound genes (2.1%) overlapped the up-regulated genes (Figure S4A). Similarly, these results were also observed in the Ssu72-depleted hepatocytes and ES cells (Figure S4B), indicating that the Ssu72-bound genes are mainly associated with the genes down-regulated by Ssu72 depletion in the three different cell types.

GO term enrichment analysis also revealed that the GO terms of Ssu72 ChIP-Seq data highly overlapped with the GO terms of the downregulated genes in the RNA-Seq results (Figure S4C-D). This analysis supported that the cellular functionality of genes downregulated by Ssu72 depletion were remarkably diverse and dependent on the cell type. Finally, we further assessed the expression levels of the Ssu72-bound genes by ChIP-Seq analyses in the three different cell types based on the RNA-Seq data. As a result, the average transcript levels of Ssu72-bound genes were noticeably reduced in all cell types (Figure S4E). We also performed GO analysis to classify the genes up-regulated by Ssu72 depletion (Figure S5A). Importantly, it is possible that the aberrantly up-regulated genes were late-response genes to Ssu72 depletion because the majority of the up-regulated genes were indirect ncRNA and ncRNA processing genes in the MEFs, liver fibrosis-related genes in response to the liver defects in hepatocytes, and indirect hormone- and morphogenesis-related genes in ES cells (Figure S5A). Therefore, we thought that the up-regulated genes might be due to indirect or compensatory effects or both in response to the stress situation caused by Ssu72 depletion. Together, our data indicate that Ssu72 is a versatile factor that functionally interacts with multiple networks to affect the expression of highly transcribed gene subsets in a cell-type-specific manner.

As yeast Ssu72 can regulate polyadenylation-coupled termination by interacting with CPF/CPSF complexes, we could not exclude any polyadenylation or RNA processing effects on altered gene expression. Therefore, we performed the poly(A) tail length assay to measure the poly(A) tail length of multiple mRNAs in MEFs and hepatocytes. Unexpectedly, we did not observe remarkable changes to actively transcribed genes in either the control or the Ssu72-depleted cells (Figure 2F), whereas some genes, which harbored transcription end site (TES)-bound Ssu72, led to slightly reduced polyadenylation activity by Ssu72 depletion (Figure S3E). We thus conclude that mammalian Ssu72 is predominantly localized to promoter regions and is broadly associated with actively transcribed genes in a cell-type-specific manner.

### Ssu72 is required for proper Pol II pause release and elongation in actively transcribed genes

**Figure 3:**
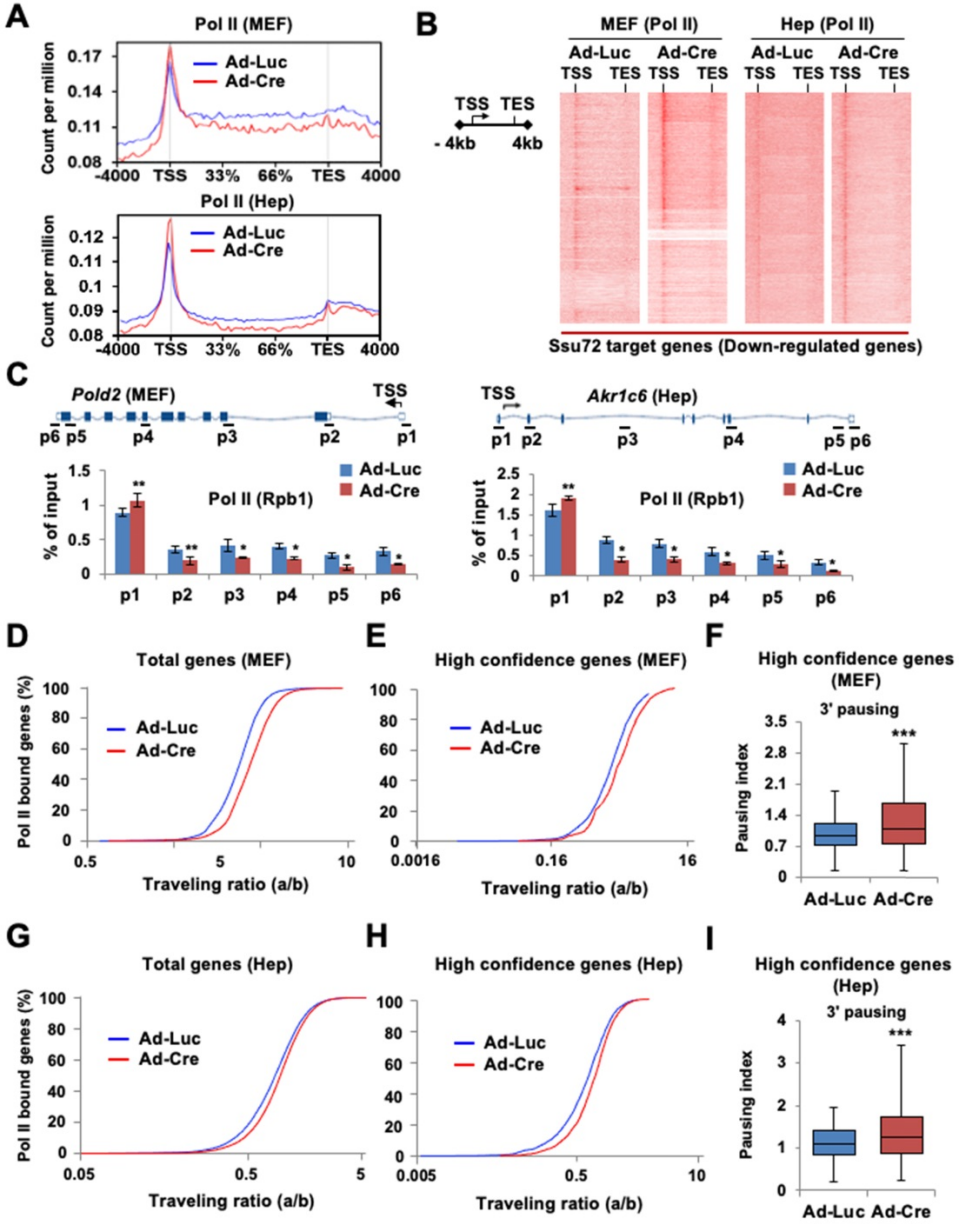
** Ssu72 is required for proper Pol II pause release and elongation in actively transcribed genes. (A)** Graphs showing high-confident profiles of genome-wide Pol II (Rpb1) occupancies in genes of Ad-Luc or Ad-Cre infected MEFs and hepatocytes. **(B)** Density plots of Pol II (Rpb1) distributions at Ssu72-bound genes (down-regulated genes by Ssu72 depletion) in control and Ssu72-depleted MEFs and hepatocytes. Each panel represents 4 kb upstream of TSS, the gene body, and 4 kb downstream of the TES. **(C)** ChIP and qPCR analyses of Pol II against Pold2 and Akr1c6 genes in control and Ssu72-depleted MEFs and hepatocytes. Data are representatives of three independent experiments. **(D)** Average genome-wide Pol II occupancy profiles showing the calculation used to determine traveling ratio (TR) in Ad-Luc and Ad-Cre-infected MEFs (also see Figure S6C). **(E)** TR of Pol II occupancy profiles against Ssu72-bound genes in Ad-Luc and Ad-Cre-infected MEFs. **(F)** Pausing index for 3'-end processing. It is the ratio of Pol II density in the transcription termination region 'c' bin to the Pol II density in the gene body region 'b' bin (also see Figure S6C). Error bars indicate SEM (*** *p* = 2.501e-29). **(G)** TR of Pol II occupancy profiles in Ad-Luc and Ad-Cre-infected hepatocytes (also see Figure S6D). **(H)** TR of Pol II occupancy profiles against Ssu72-bound genes in Ad-Luc and Ad-Cre-infected hepatocytes. **(I)** Pausing index for 3'-end processing of hepatocyte genes (also see Figure S6D).

To investigate whether Ssu72 depletion might affect the genome-wide occupancy of Pol II, we performed ChIP-Seq with antibodies against Pol II (Rpb1) in *Ssu72*^f/f^ MEFs and hepatocytes. As Ssu72 is capable of dephosphorylating both pSer5 and pSer7, we speculated that Ssu72 might regulate gene expression throughout the transcriptional initiation stage. An increase in Pol II density at the proximal promoter region is known to be correlated with a pause in transcriptional initiation 25-27. Therefore, we investigated alterations in Pol II density near the transcription start site (TSS) in Ssu72-depleted MEFs and hepatocytes. Interestingly, Ssu72 depletion caused a slight enrichment of Pol II occupancy at the promoter whereas Pol II occupancy in the gene body was modestly decreased in Ssu72-depleted MEFs compared to that in control MEFs (Figure 3A). This change in the pattern of total Pol II occupancy was most apparent in the group of Ssu72-bound genes in both MEFs and hepatocytes (Figure 3B). In addition, ChIP analyses revealed that Ssu72 depletion decreased Pol II occupancy in the DNA polymerase delta subunit 2 (*Pold2*) gene body. *Pold2* was one of selective Ssu72-bound genes in MEFs (Figure 3C, left). Similar results were observed in Ssu72-depleted hepatocytes (Figure 3C, right). Inspection of the individual Ssu72-bound transcript tracks showed that Ssu72 depletion modestly decreased Pol II occupancy in the gene bodies of the Ssu72-bound genes, including *Pold2* (Figure S6A). In contrast, Pol II occupancy of housekeeping genes such as *Rplp0* and *Hsp90* was not altered in Ssu72-deficient cells (Figure S6B).

Next, to quantitatively assess the effect of Ssu72 on Pol II occupancy in gene bodies, we calculated the traveling ratio (TR) of Pol II occupancy. This TR represents the ratio of Pol II density in the promoter-proximal region to that in the expanded gene body and also denotes the transcriptional elongation activity 26 (Figure S6C-S6D). As expected, Ssu72 depletion resulted in increased TR values for all Pol II target genes (Figure 3D), consistent with Pol II pausing in the promoter regions and decreased productive elongation. To further validate the respective ChIP-Seq peaks, the genes were classified into three groups according to peak density: high confidence, 0-30%; intermediate, 30-50%; and low, 50-100%. As expected, Pol II TR value in the group of high confidence genes was also clearly increased by Ssu72 depletion (Figure 3E). Furthermore, we calculated the transcriptional 3' pausing index for the high confidence group to assess the Pol II pausing level and elongation activity in the termination region (Figure 3F and Figure S6C). Whisker plots of the 3' pausing index in MEFs showed higher concentrations of Pol II in the 3'-end region in the Ssu72-depleted cells, implying that Ssu72 depletion slightly caused the transcriptional elongation defects in termination region of its target genes (Figure 3F). These results were also observed in Ssu72-depleted hepatocytes (Figure 3I). Taken together, these results suggest that mammalian Ssu72 may be associated with Pol II pause release and elongation to ensure accurate expression of the Ssu72 target genes, particularly the actively transcribed genes.

### Ssu72 is required for efficient transcriptional elongation in response to cellular transcription signals

**Figure 4:**
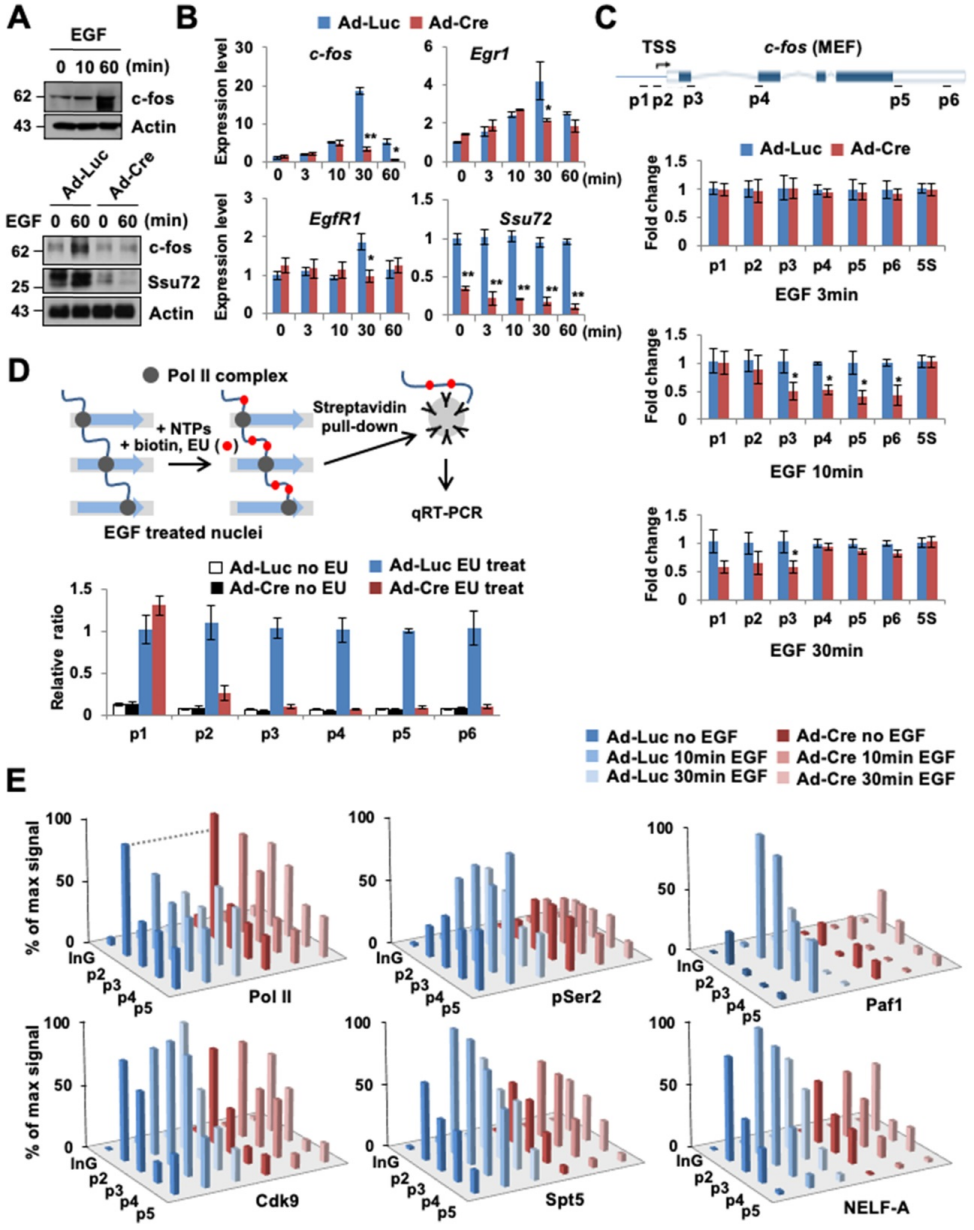
** Ssu72 is required for efficient transcriptional initiation and elongation in response to cellular transcription signals. (A)** Prior to harvest, MEFs were cultured with serum-free media for 24 h and then stimulated with 0.5 µg/mL EGF for 10 and 60 min, respectively. Cellular extracts were immunoblotted with anti-c-fos and anti-Actin antibodies (upper panel). Ad-Luc or Ad-Cre-infected MEF cell extracts (EGF: 0.5 µg/mL for 60 min) analyzed by Western blot, using indicated antibodies (bottom panel). **(B)** Total RNAs extracted from EGF-treated Ad-Luc or Ad-Cre-infected MEF cell extracts (EGF: 0.5 µg/mL for 3, 10, 30, and 60 min) analyzed by qRT-PCR using *c-fos*, Egr1, EgrR1, and Ssu72 primer sets. Error bars indicate SD. **(C)** Serum-deprived Ad-Luc or Ad-Cre-infected MEFs treated with EGF for the indicated time period and analyzed by qRT-PCR using intronic primers (p1~p6) of *c-fos* gene. 5S mRNA level was unaffected. It served as a control. Error bars indicate SD. **(D)** Nuclear run-on analyses. Serum-deprived Ad-Luc or Ad-Cre-infected MEFs were treated with 0.5 µg/mL EGF for 10 min. Transcription was stalled and then allowed to continue for 30 min in NTPs containing EU. Biotin-labeled RNAs were purified with streptavidin beads and analyzed by qRT-PCR using primers (p1 ~ p6) of *c-fos* gene. **(E)** ChIP and qPCR analyses of Pol II, pSer2, Cdk9, Spt5, Paf1, and NELF-A using primers (InG: upstream intergenic region of *c-fos* promoter and p2 ~ p5) of *c-fos* gene in EGF treated control and Ssu72-depleted MEFs (EGF: 0.5 µg/mL for 10 and 30 min). A schematic representation of *c-fos* gene and locations of primer sets for qPCR analysis are shown in Figure 4C. Data are representatives of two independent experiments.

To investigate the role of Ssu72 in the immediate response to signal-induced actively transcribed gene expression in living cells, we analyzed the mRNA expression levels of the epidermal growth factor (EGF)-responsive genes in MEFs. The *c-fos* gene is one such actively transcribed gene (a Ssu72 target gene) in MEFs regulated by mammalian Ssu72 (Figure 1E). Hence, we used EGF-inducible *c-fos* transcription as a model system and examined its mRNA and protein levels (Figure 4A-B). EGF normally stimulated c-fos protein and gene expression by more than 15-fold in serum-deprived control MEFs after EGF administration. However, EGF stimulation was significantly attenuated in Ssu72-depleted MEFs. Ssu72 depletion also decreased the expression of other EGF-responsive genes (*Egr1* and *EgfR1*) at multiple time points (Figure 4B). The accumulated occupancy of Pol II within proximal promoter and gene bodies implicates the role of Ssu72 in the transcriptional elongation process. To test this possibility, we first examined the transcription of intron-containing nascent genes through the qPCR analyses of DNase-treated total RNAs and provided specific intronic regions of the representative *c-fos* gene to monitor any potential transcriptional stress of transcriptional elongation after EGF treatment (Figure 4C). Although, we did not observe the reduction of *c-fos* expression by Ssu72 depletion within 3 min after EGF administration, our results showed a slight reduction at the beginning of *c-fos* gene transcription (p2 primer site) with dramatic attenuation in the middle and termination regions of this gene (p3 to p6 primer sites) at 10 min after EGF administration in Ssu72 depleted MEFs. At 30 min after EGF administration, the mRNA expression levels in the middle and termination regions of the *c-fos* gene were moderately restored in Ssu72 depleted MEFs, implying that Ssu72 depletion caused defects or delays in transcriptional elongation. Similar results were observed in other EGF-target genes (*c-Myc*, *Elk1* and *Ccne1*) (Figure S5B).

To further demonstrate that transcriptional elongation experienced defects after Ssu72 depletion, we performed a nuclear run-on assay, using ethylene uridine (EU) under an EGF-treated condition for 10 min (Figure 4D). After serum-deprived Ssu72 WT and KO MEFs were treated with EGF for 10 min, active transcription was stalled. Then, nuclei were isolated, followed by incubation with nucleotide triphosphates (NTPs) containing EU for 30 min. In this experiment, we found that the transcriptional elongation process of the *c-fos* gene was dramatically attenuated by Ssu72 depletion after a series of Pol II pausing events on the *c-fos* gene (Figure 4D). This result provides strong evidence that mammalian Ssu72 can positively affect the actual rate of transcription. Although Pol II still promoted transcription elongation in Ssu72-depleted cells, the pace was slower because of defective transcriptional elongation.

Given the clear effect of Ssu72 depletion on transcriptional elongation rates of actively transcribed genes, we next focused on the association of Pol II transcriptional pausing with elongation complexes. Pol II was preloaded at the *c-fos* proximal promoter. Clear elongation of Pol II was observed at gene body after EGF treatment in Ssu72 WT MEFs, whereas Ssu72 depletion resulted in stalling of Pol II in the promoter region after EGF treatment (Figure 4E). Pol II CTD phosphorylation at Ser2 is closely linked to transcriptional elongation, and Ser2 phosphorylation is typically accumulated at the 3′ ends of genes 28. Depletion of Ssu72 clearly reduced the level of Ser2 phosphorylation in all *c-fos* gene body regions (Figure 4E), indicating that Ssu72 is involved in transcriptional elongation events.

We also found that the recruitment of Cdk9, DSIF (a dimer of Spt4 and Spt5), and NELF to the *c-fos* loci was impaired by Ssu72 depletion (Figure 4E). NELF and DSIF are key regulators of Pol II pausing and releasing at promoters 29. Upon promoter escape, Spt5 associates with Pol II and functions as a positive elongation factor 30. Our ChIP analyses demonstrated that Spt5 and NELF-A occupied the *c-fos* promoter carrying preloaded Pol II. Upon activation by EGF administration, Spt5 and NELF-A were recruited strongly to the promoter and gene body in Ssu72 WT MEFs whereas those in Ssu72 depleted MEFs were very slightly recruited (Figure 4E). Such observations might reflect a defect of Paf1 recruitment because association of Paf1 with the transcription complex was influenced by recruitment and phosphorylation status of Spt5 (Figure 4E). In addition, Ssu72 depletion resulted in interference of NELF dissociation in the promoter after EGF treatment, implying that the persistent occupation by preloaded NELF complex in the promoter might result in the defect and/or delay of Pol II pausing and release (Figure 4E). These results were also observed in Ssu72-depleted hepatocytes when actively transcribed gene (*Akr1c6*) was stimulated with H_2_O_2_ treatment (Figure S5C), indicating that Ssu72 could orchestrate key events in the formation of a functional elongation complex for the accurate Pol II pause release and elongation.

### Ssu72 depletion leads to hyper-phosphorylation of CTD Ser5 and Ser7 in all regions of the promoter, gene body, and 3'-end with concomitant hypo-phosphorylation of Ser2 and Thr4 in the gene body

**Figure 5:**
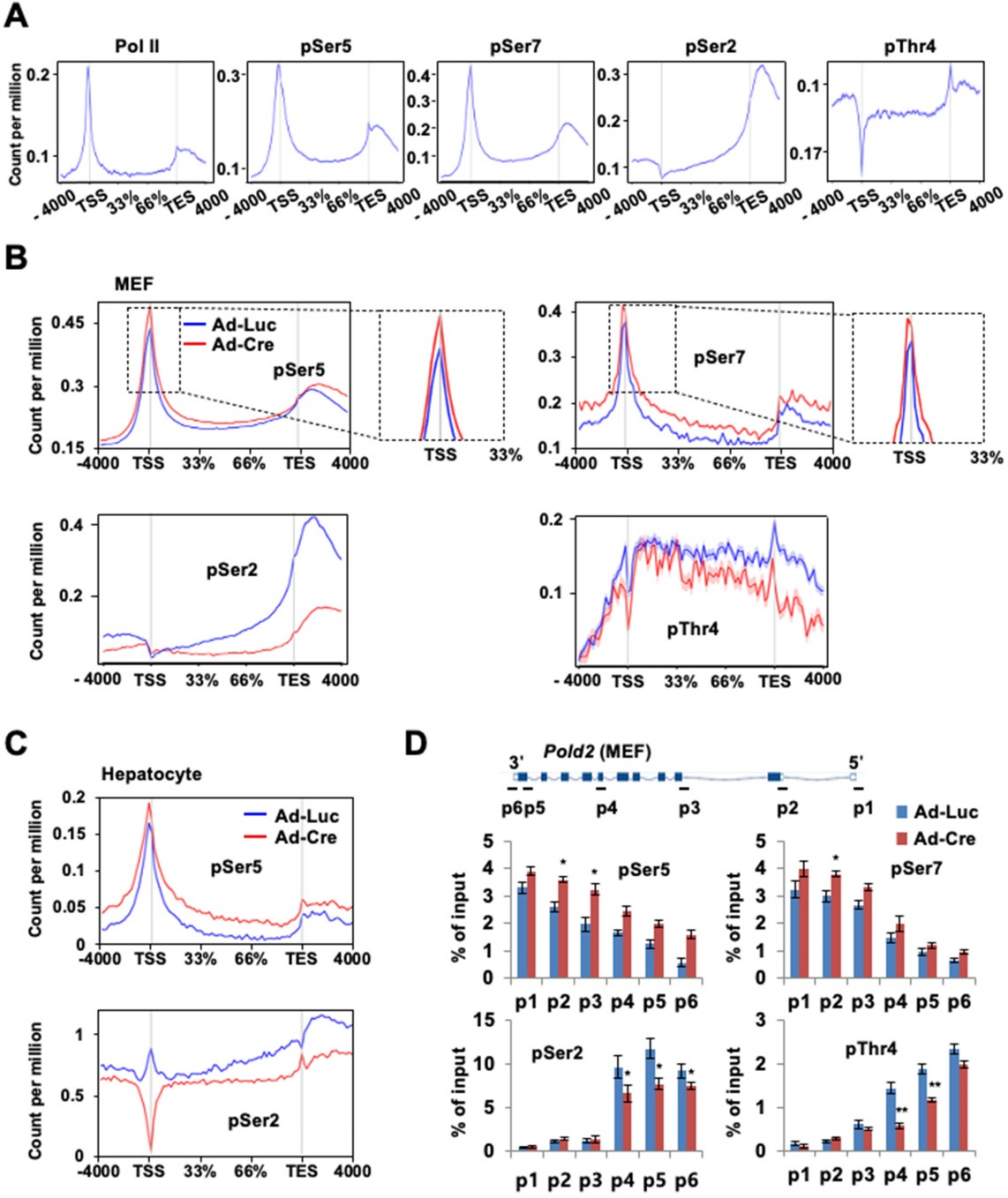
** Ssu72 depletion alters phosphorylation pattern of CTD with hyperphosphorylation of Ser5 and Ser7 and hypo-phosphorylation of Ser2 and Thr4 residues. (A)** Representative genome-wide occupancy profile of Pol II and Pol II phosphorylation (pSer5, pSer7, pSer2, and pThr4) in control MEFs. **(B)** The gene occupancies of pSer5-, pSer2-, pSer7-, and pThr4-Pol II phosphorylation in control (Ad-Luc) and Ssu72-depleted (Ad-Cre) MEFs. The right panel showing pSer5- and pSer7-Pol II occupancy profiles is an enlarged view between TSS and the initially transcribed region (33%). **(C)** The gene occupancies of pSer5- and pSer2-Pol II phosphorylation in control (Ad-Luc) and Ssu72- depleted (Ad-Cre) hepatocytes. **(D)** ChIP and qPCR analyses of pSer5, pSer7, pSer2, and pThr4 against Pold2 gene in control and Ssu72-depleted MEFs. A schematic representation of mouse Pold2 gene and locations of primer sets for qPCR analysis are shown in the upper panel. Data are representatives of three independent experiments. Error bars indicate SD.

Given the relevance of Ssu72 in Pol II accumulation at the promoter and its involvement in transcription elongation, we next examined the effect of Ssu72 depletion on genome-wide phosphorylation of Pol II and Pol II CTD phosphorylation (pSer5, pSer7, pSer2, and pThr4) (Figure 5A). As expected, pSer5- and pSer7-Pol II occupancies were moderately enriched in the promoter region by Ssu72 depletion (Figure 5B, upper panel). Interestingly, pSer5- and pSer7-Pol II occupancies in the gene body regions also remained remarkably high compared to Ssu72 WT (Figure 5B, upper panel). In addition, pSer5- and pSer7-Pol II occupancies normalized to Pol II also revealed similar results (Figure S7A), indicating that Ssu72 depletion might result in defects of transcriptional elongation and Pol II pausing. Strikingly, the occupancy of Pol II pSer2 in the gene body and 3'-end region was significantly decreased by Ssu72 depletion in MEFs (Figure 5B, bottom panel). A moderate decrease was also observed for pThr4 (Figure 5B, bottom panel). These results indicate that Ssu72 depletion is directly associated with transcriptional elongation defects through a reduction in pSer2 and pThr4 levels of Pol II at the gene body. It has been reported that steady increases in both pSer2 and pThr4 in the gene body and the 3'-end region are linked to the regulation of transcriptional elongation in organisms ranging from yeast to humans 31, 32. These results demonstrate that mammalian Ssu72 may participate in effective transcriptional elongation by regulating phosphorylation of Ser2 and Thr4 residues. Similar results were also observed in all occupancies and normalized occupancies of pSer5- and pSer2-Pol II against hepatocytes (Figure 5C and Figure S7B).

Our ChIP analyses of *Pold2* and *Phyhd1* (Ssu72 target genes in MEFs) as well as *Akr1c6* (a Ssu72 target gene in hepatocytes) revealed that the levels of pSer5 and pSer7 were increased at all regions with a concomitant decrease in the levels of pSer2 and pThr4 within the gene body in Ssu72-deficient MEFs (Figure 5D and Figure S7C-D). Although we could not exclude the possibility of limitation of each phospho-CTD antibody being involved in epitope interference between phospho-CTD antibodies, our results showed dynamic changes of CTD phosphorylation caused by Ssu72 depletion.

### Ssu72 influences diverse phosphorylation patterns in CTD depending on cell type

We next performed immunoblotting to examine whether Ssu72 could regulate the phosphorylation of CTD in a cell-type-specific manner. Immunoblotting analyses revealed that the levels of pSer5 and pSer7 were slightly increased in Ssu72-deficient MEFs compared to those in wild-type MEFs. Unexpectedly, levels of pSer2 and pThr4 were markedly decreased in Ssu72-deficient MEFs (Figure S8A-B). However, we did not find any notable changes in non- and/or hypo-phosphorylated Pol II CTD (Figure S8A-B). Similarly, Ssu72-depleted hepatocytes of both Ad-Cre-infected and Alb-Cre cross-mated mice showed moderate changes in their phosphorylation patterns (Figure S8C-D). We next compared the phosphorylation profiles of CTD at the Ser2, Ser5, Ser7, and Thr4 residues between WT (*Ssu72^+/+^*) and Ssu72-deficient (*Ssu72^∆/∆^*) embryos. The results of the immunostaining analyses using blastocysts revealed that *Ssu72^∆/∆^* embryonic cells showed a clear increase in phosphorylated forms of CTD at Ser5 and Ser7 (average intensities of pSer5 and pSer7: 1,427 and 1,023 AU, respectively) compared to the *Ssu72^+/+^* embryonic cells (average intensities of pSer5 and pSer7: 905 and 639 AU, respectively) (Figure S8E). By contrast, the levels of pSer2 and pThr4 were dramatically reduced in *Ssu72^∆/∆^* embryonic cells compared to those in *Ssu72^+/+^* embryonic cells (average intensities of pSer2 and pThr4: 1,023 and 896 AU in *Ssu72^+/+^* embryonic cells, and 639 and 455 AU in *Ssu72^∆/∆^* embryonic cells, respectively) (Figure S8E). Taken together, these results suggest that Ssu72 can regulate inverse phosphorylation patterns between CTD states (hyperphosphorylation of Ser5 and Ser7 and hypophosphorylation of Ser2 and Thr4).

### Direct coupling between hyper-phosphorylation of Ser5 and Ser7 and the hypo-phosphorylation of Ser2 and Thr4 by Ssu72 depletion

To further compare the phosphorylation profiles of each CTD residue, we immunoprecipitated the Pol II complex with an anti-pSer5 antibody. High levels of pSer5 and pSer7 were detected in the immuno-complex in Ssu72-depleted MEFs, whereas pSer2 and pThr4 levels in the pSer5-mediated immuno-complex were significantly decreased in the Ssu72-depleted MEFs compared to the control MEFs (Figure S9A). Consistently, levels of pSer5 and pSer7 in the pSer2 immuno-complex were increased in Ssu72-depleted cells (Figure S9A). These results suggest that induced hyper-phosphorylation of Ser5 and Ser7 residues by Ssu72 depletion may be directly linked to hypo-phosphorylation of Ser2 and Thr4 residues of CTD codes.

We next questioned whether overexpression of Ssu72 phosphatase could affect the change of phosphorylation profile in CTD codes. Interestingly, overexpression of Ssu72 wild-type (WT) in MEFs caused reductions in pSer5 and pSer7 levels, whereas the levels of pSer2 and pThr4 were almost unaffected by Ssu72 overexpression. As a negative control, the overexpression of an Ssu72 phosphatase‐dead mutant (Ssu72 C12S) showed very similar patterns of phosphorylation profiles of CTD codes to the control (Figure S9B), indicating that Ssu72 directly affects the phosphorylation of Ser5 and Ser7 residues but not that of Ser2 and Thr4 residues. In addition, we examined whether re-introduction of Ssu72 into the Ssu72-deficient MEFs could rescue the hypo-phosphorylation of pSer5 and pSer7 residues. Ssu72-deficient MEFs were transfected with an expression plasmid encoding a control tandem affinity purification (TAP) tag, TAP-tagged Ssu72 WT (TAP-Ssu72 WT), or TAP-tagged Ssu72 C12S (TAP-Ssu72 C12S) phosphatase‐dead mutant (Figure S9C). As expected, the levels of pSer5 and pSer7 were significantly decreased by the overexpression of TAP-Ssu72 WT but not TAP-Ssu72 C12S (Figure S9C). However, the levels of pSer2 and pThr4 were also unaffected by Ssu72 complementation, indicating that the Ser2 and Thr4 residues may not be the direct substrates of Ssu72 phosphatase. In summary, these results suggest that Ssu72 phosphatase directly regulates the hyperphosphorylation of Ser5 and Ser7 and, moreover, is indirectly involved in regulating the hypophosphorylation of Ser2 and Thr4 residues. To further characterize the phosphorylation states of each CTD residue in MEFs, we performed 2-dimensional isoelectric focusing (IEF) gel electrophoresis using cellular lysates of adenovirus-luciferase (Ad-Luc)-infected or Ad-Cre-infected Ssu72^f/f^ MEFs. Immunoblotting with anti-phospho-CTD antibodies revealed that depletion of Ssu72 led to an acidic shift of pSer5- and pSer7-containing polypeptides, confirming the hyperphosphorylation of Ser5 and Ser7 residues by Ssu72 depletion (Figure S9D). We also observed that the levels of pSer2- and pThr4-containing polypeptides were significantly reduced, as they were shifted to a more basic isoelectric point in Ssu72-deficient MEFs compared to WT MEFs (Figure S9D). As a result, we showed that mammalian Ssu72 regulates the inverse phosphorylation pattern between CTD codes (hyperphosphorylation of Ser5 and Ser7 and hypophosphorylation of Ser2 and Thr4).

### Ssu72 is required for activation and recruitment of positive transcription elongation factor b (P-TEFb) in transcriptional elongation

**Figure 6:**
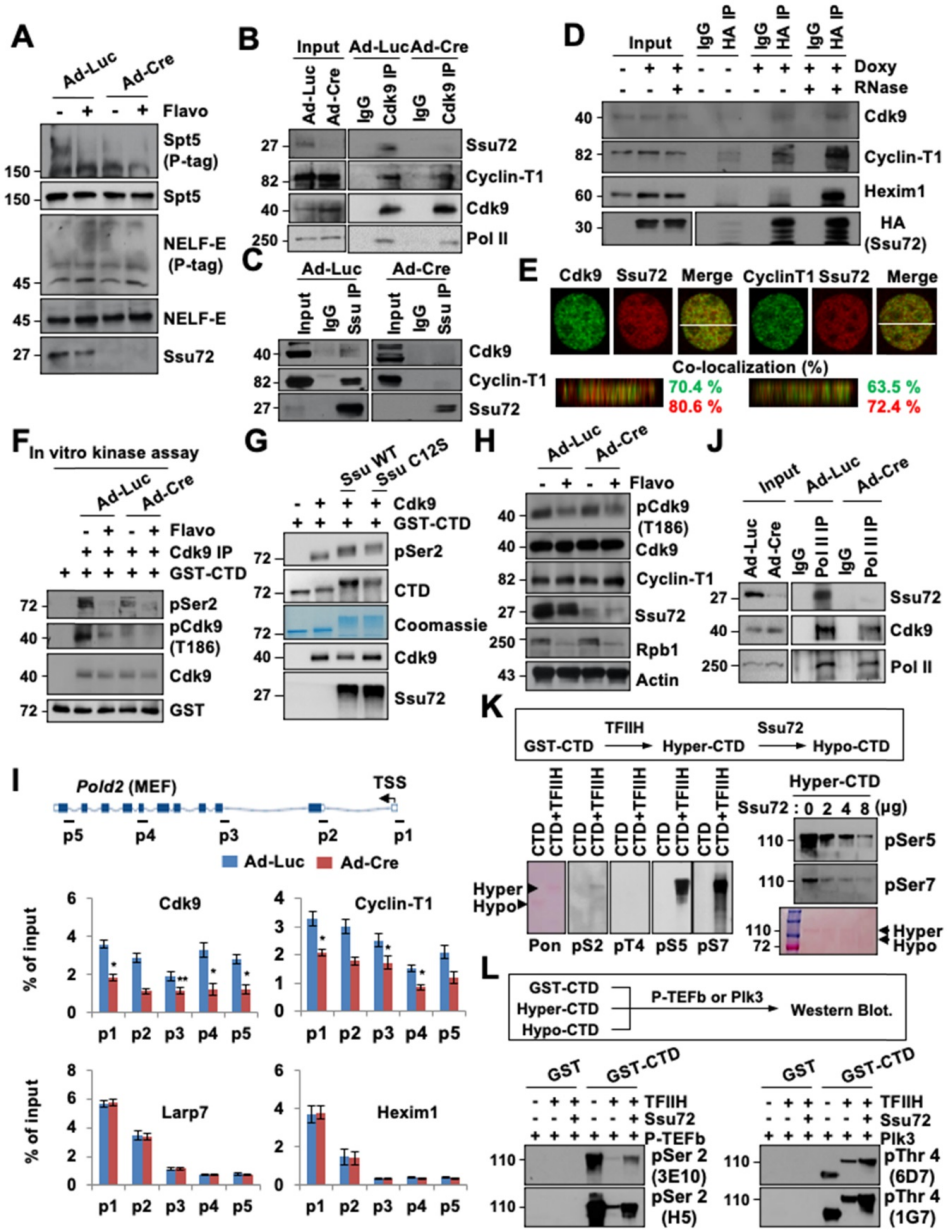
** Ssu72 is required for activation and recruitment of P-TEFb to regulate accurate transcriptional elongation. (A)** Immunoblotting of Ad-Luc or Ad-Cre-infected MEF cell lysates. Phos-tag denotes Phos-tag gel used to resolve phosphorylation based on mobility shift. **(B and C)** Extracts from Ad-Luc or Ad-Cre-infected MEF cells immunoprecipitated with IgG, anti-Cdk9, or anti-Ssu72 antibodies. **(D)** Inducible MEFs expressing HA-Ssu72 cultured in the absence (-) or presence (+) of 2 µg/ml doxycycline for 48 h. Cellular extracts were incubated in the absence (-) or presence (+) of 50 µg/ml RNAase (RNase), and immunoprecipitated with IgG or HA antibodies. **(E)** MEF cells stained with indicated antibodies. Percentages of co-localization between green and red signals were calculated by orthogonal section analyses (ZEN software). **(F)** Ad-Luc or Ad-Cre-infected MEFs were cultured in the absence (-) or presence (+) of 500 nM Flavopiridol for 8 h. Lysates were immunoprecipitated with anti-Cdk9 antibody. Immunocomplexes were incubated with GST-CTD (1 μg) in the presence of cold ATP and analyzed by immunoblotting. **(G)** Purified GST-CTD was incubated with recombinant Cdk9 and eluted Ssu72 proteins (His-Ssu72 WT or His-Ssu72 C12S) in the presence of cold ATP. The reaction was stopped by the addition of SDS sample buffer and immunoblotted with indicated antibodies. **(H)** Ad-Luc and AdCre-infected MEF cell lysates immunoblotted for phosphorylation status. **(I)** ChIP and qPCR analyses of Cdk9, Cyclin T1, Larp7, and Hexim1 against Pold2 gene in control and Ssu72-depleted MEFs. **(J)** Extracts from Ad-Luc or Ad-Cre-infected MEFs immunoprecipitated with IgG and anti-Pol II antibodies. Immunocomplexes were analyzed by Western blotting using indicated antibodies. **(K)** GST-CTD protein-bound beads were incubated with recombinant TFIIH complex in the presence of cold ATP, stained with Ponceau S, and immunoblotted with indicated antibodies. **(L)** GST, GST-CTD, hyper-CTD, and hypo-CTD proteins were incubated with recombinant P-TEFb or Plk3 kinase in the presence of cold ATP and immunoblotted with pSer2 and pThr4 antibodies.

Many effects of Ssu72 depletion on Pol II transcriptional activity at the elongation factor recruitment step could be explained by decreased recruitment of P-TEFb. Previous studies have demonstrated that productive elongation begins with the recruitment of P-TEFb, which phosphorylates NELF and DSIF, resulting in a release of the transcriptional pause 30,33. To examine the potential crosstalk between Ssu72 and P-TEFb activity, Ad-Luc-infected and Ad-Cre-infected MEFs were cultured in the absence or presence of 500 nM flavopiridol, an inhibitor of the phosphorylation of specific substrates, including the phosphorylation of CTD pSer2 and pThr4 residues by P-TEFb (a Cdk9 kinase). As expected, flavopiridol remarkably reduced Spt5 phosphorylation, with a mobility shift on a Phos-tag (p-tag) gel (Figure 6A). Surprisingly, Ssu72 depletion in MEFs also significantly reduced phosphorylation of Spf5 regardless of flavopiridol treatment, while NELF phosphorylation levels were not altered (Figure 6A).

This raises the possibility that Ssu72 may regulate kinase activity of P-TEFb, thereby contributing to phosphorylation of DSIF and Ser2 residues in CTD for effective transcriptional elongation. To test this possibility, we first examined whether direct interactions between Ssu72 and P-TEFb were involved in the Cdk9-Cyclin T1 complex. Cell extracts were immunoprecipitated with Cdk9 and Ssu72 antibodies or normal IgG. Immunoblotting was then performed with antibodies against Ssu72, Cdk9, Cyclin T1, and Pol II (Figure 6B-C). Notably, Ssu72 and P-TEFb were present in the complexes in vivo, although Ssu72 was more strongly bound to Cyclin T1 than to Cdk9 (Figure 6C), implying that Ssu72 might form a complex with P-TEFb through Cyclin T1 protein.

To further validate that Ssu72 could interact with the P-TEFb complex, we performed immunoprecipitation analyses using inducible HA-Ssu72 MEFs. Our immunoprecipitation analyses revealed that HA-Ssu72 protein formed a complex with endogenous Cdk9 as well as with Cyclin T1 and Hexim1 (Figure 6D). Interestingly, the interaction between Ssu72 and the P-TEFb complex was largely insensitive to RNase (Figure 6D) as expected for a protein-protein complex interaction, implying that Ssu72 might not bind to P-TEFb through the 7SK snRNP complex. Additionally, we further performed the proteomic analyses against immuno-complexes of Ssu72 and discovered the various proteins such as NcoR1, Sart1, Hexim and DDX5 as a component of the P-TEFb complex (Table S6). Furthermore, we performed 3D high-resolution confocal microscopy of Ssu72 and the components of the P-TEFb complex and observed significant co-localization of their protein pair combinations in MEFs (Figure 6E). To further validate the relevance of these findings, we assessed the kinase activity of P-TEFb by immunoprecipitating the P-TEFb complex using an anti-Cdk9 antibody under control and Ssu72-depletion conditions (Figure 6F). Purified GST-CTD proteins were incubated with cold ATP to immunoprecipitate the P-TEFb complexes (Figure 6F). While both the control and the Ssu72-depleted cells showed significantly decreased P-TEFb kinase activity after flavopiridol treatment, the kinase activity of P-TEFb was slightly reduced in Ssu72-depleted cells without flavopiridol treatment (Figure 6F), implying that Ssu72 might regulate the kinase activity of P-TEFb. We next examined the *in vitro* kinase assay of P-TEFb using recombinant Ssu72 WT and Ssu72 C12S. Interestingly, both recombinant Ssu72 WT and C12S were significantly increased the kinase activity of P-TEFb, implying that the P-TEFb kinase activity might be induced by the protein interaction between Ssu72 and P-TEFb rather than the Ssu72 phosphatase activity (Figure 6G). We also examined the phosphorylation level of Cdk9 at Thr 186 residue using immunoblotting and ChIP analyses to further assess P-TEFb activity under Ssu72 depleted condition (Figure 6H and Figure S10A). However, we did not observe significant phosphorylation changes of Cdk9 at Thr186 residue.

Given that Ssu72 is associated with the P-TEFb complex and that both of them are recruited to chromatin, we hypothesized that Ssu72 could recruit the P-TEFb complex to chromatin for effective Pol II pause release and elongation. To determine whether Ssu72 depletion could affect chromatin loading of P-TEFb, we performed Cdk9, Cyclin-T1, La-related protein 7 (Larp7), and Hexim1 occupancy ChIP analyses. Interestingly, recruitment of the P-TEFb complex onto chromatin was markedly reduced in Ssu72-depleted cells whereas chromatin binding levels of the 7SK snRNP complex (Larp7 and Hexim1) were almost unaffected by Ssu72 depletion (Figure 6I, Figure S11A). In addition, the level of binding between Cdk9 and Pol II and between Cdk9 and Pol II pSer2 was decreased by Ssu72 depletion (Figure 6J and Figure S10B). We finally assessed whether hyper-phosphorylation of the Ser5 and Ser7 residues caused by Ssu72 depletion affected the recruitment of P-TEFb. To induce hyper-phosphorylation of the Ser5 and Ser7 residues (“hyper”-CTD), the GST-CTD beads were first incubated with the TFIIH complex (containing Cdk7-cyclin H kinase) in the presence of cold ATP. After extensive washing to remove any excess TFIIH complex, the “hyper”-CTD beads were then incubated with Ssu72. As expected, hyper-phosphorylation of Ser5 and Ser7 residues by the TFIIH complex was remarkably reduced by the addition of Ssu72 phosphatase in a dose-dependent manner (“hypo”-CTD; Figure 6K). We next collected GST-CTD, “hyper”-CTD, and “hypo”-CTD beads and incubated them with P-TEFb complex or Plk3 kinase (a specific CTD Thr4 residue kinase). Intriguingly, hyper-phosphorylation of Ser5 and Ser7 residues (“hyper”-CTD) by the TFIIH complex significantly reduced P-TEFb-mediated Ser2 and Plk3-mediated Thr4 phosphorylation of GST-CTD (Figure 6L), implying that hyper-phosphorylation of Ser5 and Ser7 by the TFIIH complex could directly affect the hypo-phosphorylation of Ser2 and Thr4 by inhibiting the P-TEFb complex and Plk3 kinase recruitment to CTD. These data indicate that mammalian Ssu72 is involved in the transcriptional initiation and elongation processes by regulating the recruitment and activity of the P-TEFb complex that can induce elongation through phosphorylation of the CTD Ser2 and Thr4 residues (Figure S11B). Therefore, mammalian Ssu72 is directly associated with accurate expression of downstream target genes, particularly actively transcribed genes, by regulating Pol II pause release and elongation.

## Discussion

Ssu72 is highly conserved from yeast to mammals. It is essential for yeast cell viability and an efficient transcriptional process 12, 20. However, the details of how Ssu72 contributes to the gene expression process by modulating the transcriptional cycle in mammalian cells remain unclear. Our study demonstrated that the underlying mechanism of action of mammalian Ssu72 involved coordination of hyper-phosphorylation and hypo-phosphorylation of cell type-specific CTD residues. Ssu72 deficiency resulted in constitutive hyper-phosphorylation of CTD Ser5 and Ser7 residues in the promoter region, although this effect was dependent on cell type. These findings indicate that hyper-phosphorylation of CTD at the Ser5 and Ser7 residues by Ssu72 depletion can lead to local accumulation of Pol II within a promoter-proximal region. These results also imply that mammalian Ssu72 is a critical component of Pol II pause release and productive elongation.

Unexpectedly, this study also provided evidence that mammalian Ssu72 was associated with both direct and indirect mechanisms of phosphorylation dynamics of the CTD Ser2 and Thr4 residues through regulation of P-TEFb activity and recruitment on chromatin during transcriptional elongation. A principal mechanism by which P-TEFb influences transcriptional events is phosphorylation of the Pol II CTD Ser2 and Thr4 residues to recruit factors that regulate elongation, RNA processing, and chromatin modification 34. In the present study, Ssu72 directly interacted with the P-TEFb complex and regulated the kinase activity of P-TEFb. In addition, hyper-phosphorylation of CTD Ser5 and Ser7 residues by Ssu72 depletion seemed to be closely associated with hypo-phosphorylation of the CTD Ser2 and Thr4 residues, and *vice versa*. This effect is likely to be linked to inhibition of P-TEFb and elongation factor (DSIF) recruitment onto the gene body regions, thereby causing severe defects in transcriptional elongation during Pol II-mediated gene expression of Ssu72-bound genes (actively transcribed genes).

During transcription elongation, the CTD phosphorylation state is highly dynamic. It takes place within a short time, almost simultaneously. During this process, the levels of pSer5 and pSer7 are sharply decreased whereas pSer2 levels are increased across gene bodies 1. Although these phosphorylation patterns are not coincidental, when and how P-TEFb could recognize a specific CTD state in promoter regions remain unknown. Additionally, it is impossible that hyper-phosphorylation of CTD Ser5 and hypo-phosphorylation of CTD Ser2 might appear at the same time and region in the current model. However, our model makes it possible to explain this particular state (hyper-phosphorylation of CTD Ser5 and hypo-phosphorylation of CTD Ser2 in the same region) through the interaction between Ssu72 and P-TEFb (Figure S11B). These observations suggest that mammalian Ssu72 functions as a *bona fide* protein phosphatase that can regulate finely-tuned Pol II pause release and active elongation.

Recent reports on Thr4 phosphorylation required for transcriptional elongation of protein-coding genes 32 suggest that Pol II CTD pThr4 may play a critical role in a specific histone gene expression program 35, 36. Those reports have also shown that hyper-phosphorylation of Thr4 is required for effective mRNA 3'-end processing and histone gene expression, thus facilitating recruitment of 3' processing factors to histone genes 35, 36. Interestingly, our study provided novel evidence that Ssu72 could regulate hyper-phosphorylation of the Thr4 residue in CTD, indicating that transcriptional elongation defects caused by Ssu72 depletion were also associated with dysregulation of CTD Thr4 phosphorylation. In particular, depletion of Ssu72 resulted in a sharp reduction of pThr4 in ES cells. Moreover, expression levels of chromatin assembly-related genes, including a histone gene cluster, were remarkably decreased in Ssu72-deficient ES cells (Table S1), in agreement with a previous report 35. This indicates that Ssu72 is also associated with accurate transcription of histone genes by regulating CTD Thr4 phosphorylation in an ES cell-specific manner. Taken together, these findings indicate that Ssu72 deficiency can result in hyper-phosphorylation of the CTD Ser5 and Ser7 residues in all regions of the promoter, gene body, and 3'-end with hypo-phosphorylation of CTD Ser2 and Thr4 residues in the gene body and 3'-end regions, thereby impairing transcription in transcriptionally active Ssu72-bound genes (Ssu72 target genes) in a cell-type-specific manner. However, we cannot exclude the possibility that Ssu72 might exert additional effects on hypo-phosphorylation of transcription factors and coactivators involved in the regulation of tissue-specific gene expression.

During the development and maintenance of multicellular organs, each type of cell acquires its specific fate through precise control of gene expression. Some researchers have reported that this process is mediated by tissue-specific functions of broadly expressed transcription factors as well as spatial contacts between a distal enhancer and a proximal promoter 37, 38. Interestingly, our RNA-Seq analyses revealed that deficiency of Ssu72 downregulated expression levels of distinct panels of genes involved in cell-selective functions such as cell adhesion and membrane-mediated growth signaling in primary MEFs, metabolic processes in hepatocytes, and chromatin and nucleosome assembly in ES cells. Furthermore, we found that loss of Ssu72 could induce aberrant proliferation of quiescent hepatocytes whereas Ssu72 depletion in highly proliferative MEF and ES cells caused severe growth defects (Figure S1 and Figure S12). These results imply that Ssu72-mediated CTD phosphorylation is associated with cellular and tissue homeostasis by regulating gene expression.

If so, how does mammalian Ssu72 phosphatase control the expression of distinct sets of genes in a cell-type-specific manner? One possible explanation is that Ssu72 has distinct localization patterns in various cell types, including quiescent and proliferating cells. During the transcription cycle, many CTD-specific kinases and phosphatases are recruited to Pol II CTD. The dynamics of hyper-phosphorylation and hypo-phosphorylation is a major regulatory mechanism involved in CTD modification. Various phosphatases and kinases also have different specificities toward isomeric states of proline residues adjacent to various phosphorylation sites. According to recent reports, Ssu72 can selectively recognize the *cis* configuration of the Ser5-Pro6 motif within CTD through the *cis/trans* conversion activity of Pin1 isomerase, different from other CTD phosphatases 16, 39.

In line with this evidence, Ssu72 may localize with Pin1 at the *cis* conformation of Pol II CTD. This complex is likely to affect the transcriptional machinery and mRNA expression of actively transcribed genes depending on cell type. Our ChIP-Seq results showed that Ssu72 occupied core promoters of a substantial proportion of actively transcribed and bivalent genes in all cell types tested. However, the density of the Ssu72 signal in each cell type was slightly different. The occupancy of Ssu72 was significantly increased at the 3'-end regions as much as in the promoter regions of ES cells. However, it was mostly observed in the promoter regions of MEFs and hepatocytes. Therefore, Ssu72 might be specifically localized in a distinct *cis* configuration of Pol II for actively transcribed genes in each cell type, thereby regulating different sets of transcribed genes in different cell types.

Another possibility is that Ssu72 may regulate the expression of cell type-specific gene families containing bidirectional genes by controlling gene loops. Recent reports have suggested that gene loops may bring distal regulatory elements such as enhancer and 3'-end processing components into proximity to promoter regions 40-42. Such gene loops found in actively transcribed genes can promote gene expression by allowing interactions between promoters and terminators 42. In addition, these looping interactions are specific to various cell types. They can regulate chromatin features and accurate gene expression 43. Ssu72 phosphatase has recently been implicated in the formation and maintenance of gene loops by co-associating with both promoter and terminator 44. In addition, inactivation of individual gene loops by Ssu72 depletion can enhance ncRNA synthesis from bidirectional promoters 44. Our RNA-Seq data showed that Ssu72 depletion in MEFs significantly increased ncRNA processing genes and ncRNA levels (Figure 1D). Therefore, interference with cell type-specific gene looping by Ssu72 depletion might give rise to distinct gene expression patterns in various cell types from Ssu72 knockout mice.

Altogether, this study demonstrates that mammalian Ssu72 can mediate tissue-specific transcriptional activity by regulating reciprocal phosphorylation of CTD both *in vitro* and *in vivo*. It also shows that Ssu72 deficiency can cause aberrant Pol II pausing and elongation defects during the transcriptional cycle, with diversity dependent upon the cell type based on gene transcription.

## Methods

### Generation of Ssu72 knockout mice

Mouse Ssu72 genomic DNA containing exon 1 and exon 2 was obtained from bacterial artificial chromosome (BAC) library. To construct the targeting vector for Ssu72, a single *loxP* site was inserted into exon 1, and a puromycin selection cassette flanked by FRT sites with a single *loxP* site was inserted between exons 1 and 2 (Figure S1A). The targeting construct was transfected into embryonic stem (ES) cells via electroporation. After selection with puromycin, homologous recombinants were screened by Southern blotting using a 3′ external probe. Generation of targeted ES cell clones, germ line transmission of the *Ssu72*puro allele, and generation of *Ssu72*f and *Ssu72*∆ alleles were performed. The primers used for mouse genotyping PCR are summarized in Table S7. This study was reviewed and approved by the Institutional Animal Care and Use Committee (IACUC) of National Cancer Center and Sungkyunkwan University School of Medicine.

### *In vitro* culture of pre-implantation embryos and immunostaining of embryos

For *in vitro* culture of pre-implantation embryo, blastocysts were collected on E3.5 by flushing the uterus of each mouse with M2 medium, followed by culturing in conditioned ES media (DMEM, 15% FBS, 2 mM L-glutamine, 50 units/ml penicillin, 50 μg/ml streptomycin, 55 μM 2-mercaptoethanol, and 1000 units/ml of leukemia inhibitory factor). For immunostaining of mouse embryos, blastocysts were rinsed with PBS and fixed with fresh 4% formaldehyde in PBS for 30 min at room temperature. DNA was denatured after permeabilization with 2 M HCl and 0.5% Triton X-100 for 20 min at RT. After washing extensively with PBS containing 1.0% BSA, blastocysts were collected and incubated with the indicated primary and secondary antibodies. Slides were stained with DAPI, washed with PBS, air-dried, mounted, and processed for confocal microscopy. Fluorescence intensity levels of each embryo were quantified using ZEN software.

### Immunostaining and microscopy

For immunostaining, cells were cultured directly on glass coverslips, washed with PBS, fixed in 4% paraformaldehyde, incubated with indicated primary and fluorescent-labeled secondary antibodies (Alexa Fluor 488 and 568, 1:300), and then stained with DAPI. Images were acquired on a LSM510 META confocal microscope fitted with a ×20 NA 0.75 objective lens and an AxioCam camera. Z series of 0.3 μm stacks were acquired using AxioVision Release 3.x software with a *Z*-stack module. Deconvolution was performed using Huygens software.

### Immunohistochemistry

For histological analysis, liver tissues were fixed with formalin overnight at 4 °C and embedded in paraffin, and cut into 4 μm sections. After deparaffinization and dehydration, antigen retrieval was performed by boiling the sections in 10 mM citric acid buffer (pH 6.0) for 15 min, and then cooled to room temperature for 30 min. Immunohistochemistry was performed with anti-BrdU and anti-Ki67 antibodies. For BrdU immunofluorescence staining, 5-bromo-2'-deoxyuridine (BrdU) (Sigma-Aldrich) was administered to the mice by intraperitoneal injection 2 h before harvesting the livers. Paraffin-embedded liver sections were stained with anti-BrdU antibody, DAPI solution and anti-Alexa Fluor 488 (Invitrogen Life Technologies). Images were acquired on an AxioImager M2 microscope (Carl Zeiss) with AxioVision 4.82 software (Carl Zeiss) and analyzed for hepatocyte nuclei numbers and sizes using NIH ImageJ software.

### Cell infection with recombinant adenovirus

One day prior to recombinase adenovirus infection, MEFs, hepatocytes, and ES cells were plated onto dishes. On the next day, the cells were infected with Ad-Luc or Ad-Cre in low-FBS serum containing 100 nM protein transduction domain (PTD) peptide (Sequence: GLNGPDIYKGVYQFKSVEFD) to enhance the efficiency of adenoviral infection.

### Isolation of MEFs and hepatocytes

MEFs were isolated from embryonic day 13.5 embryos according to a standard protocol. Each embryo was dissected in 10 ml sterile PBS and sheared through an 18G syringe in the presence of 0.25% trypsin and 1 mM EDTA. After 10 min of incubation at 37 ºC with gentle shaking, the cells were plated onto dishes and maintained at 37 ºC. Adherent cells were used as MEFs. Hepatocytes were isolated from 5-week-old *Ssu72*^f/f^ and Alb-Cre;*Ssu72*^f/f^ (KO) mice by liver perfusion with collagenase IV. After isolation, the hepatocytes were seeded in M199 medium supplemented with 10% FBS, 23 mM HEPES, and 10 nM dexamethasone.

### RNA-sequencing and bioinformatics analyses

Total RNA from Ad-Luc or Ad-Cre infected MEFs, hepatocytes, and ES cells were extracted with Trizol reagent and subjected to RNA-Seq analyses**.** We added 1 μL of a 1:50,000 dilution of ERCC RNA spike‐in mix (Thermo Fisher) to all samples prior to library preparation. The transcriptome sequencing libraries were prepared using TruSeq® Stranded Total RNA Library Prep Kit (Illumina) by following the manufacturer's procedure. RNA-seq libraries were sequenced on an Illumina HiSeq 2000 or 3000/4000 using paired-end with read lengths of 150 bp. An initial sequence-level quality assessment was performed using FastQC. The Fastq files with 150 bp paired-end reads were processed using Partek Flow. Briefly, the raw reads were aligned and mapped to the mouse *mm*10 reference genome using Tophat. The aligned reads are quantified to an annotation model through Partek E/M. The normalization method used here is counts per million (CPM) through Partek Flow. The normalized counts were then subjected to statistical analysis using ANOVA. Differential gene expression analysis was performed using the package DESeq2. Read counts were used and modeled based on a negative binomial distribution. We considered genes as differentially expressed if: (1) the FDR was less than 0.05, (2) the expression ratio among three independent samples was > 2-folds change, (3) there was agreement with DESeq2. Data were obtained from three independent experiments and processed using DAVID Bioinformatics Resources 6.7 software for Gene ontology, BIOCARTA, Cytoscape, KEGG pathway and GSEA.

### Immunoblot assay

For immunoblot assays, primary cells were harvested by scraping and then resuspended in TNN buffer [50 mM Tris-HCl (pH 7.5), 150 mM NaCl, 1% NP40, 1 mM phenylmethylsulfonyl fluoride (PMSF), 1 mM dithiothreitol (DTT)] containing a protease inhibitor cocktail followed by brief sonication. Equal amounts of proteins from each sample were separated by SDS-PAGE, transferred to a nitrocellulose membrane, blocked, and analyzed by immunoblotting with appropriate antibodies (Table S8).

### Immunoprecipitation, *in vitro* kinase, IP-kinase, and *in vitro* phosphatase analyses

For immunoprecipitation using total cell extracts, Ad-Luc- and Ad-Cre-infected MEF cells were resuspended in buffer A (100 mM Tris-HCl (pH 7.5), 20 mM EDTA, 1% NP40, 1 mM PMSF, 1 mM DTT, and a protease inhibitor cocktail). The supernatants (soluble cytoplasmic fractions) were obtained. The cell pellets were resuspended in buffer B [100 mM Tris-HCl (pH 7.5), 20 mM EDTA, 300 mM NaCl, 1% NP40, 1 mM PMSF, 1 mM DTT, and a protease inhibitor cocktail], briefly sonicated, and centrifuged to obtain the supernatants (soluble pellet fractions). Mixed extracts (soluble cytoplasmic and pellet supernatants) were diluted with a salt‐free buffer to reduce salt concentration to 150 mM. These samples were centrifuged and analyzed by immunoprecipitation. For the *in vitro* kinase assay, purified GST-fused CTD proteins were incubated with kinase buffer (20 mM HEPES, 1 mM EDTA, 10 mM MgCl_2_, 1 mM DTT, 200 μM ATP), and 0.5 μg of recombinant TFIIH, P-TEFb, and Plk3 kinase at 30 ºC for 1 h. The reaction was halted by addition of SDS sample buffer, resolved by SDS-PAGE, and visualized by Ponceau S staining and immunoblotting analyses. For P-TEFb IP-kinase assay, total cell lysates prepared from MEFs were incubated with Cdk9 antibody and Protein A/G agarose beads to obtain immuno-P-TEFb complexes. Purified GST-CTD (1 μg) was used as a substrate of the P-TEFb precipitate in subsequent kinase reaction as described above. For the phosphatase analyses, purified GST-Ssu72 proteins were cleaved by thrombin digestion and then incubated in a phosphatase buffer (20 mM HEPES, 1 mM EDTA, 10 mM MgCl_2_, and 1 mM DTT) containing hyper-phosphorylated GST-CTD substrates.

### Poly(A) tail length analyses by GI-tailing

Total RNAs from Ad-Luc or Ad-Cre infected MEFs were extracted with Trizol reagent and subjected to G/I tailing using Poly(A) Tail Length Assay Kit following the manufacturer's protocol. The Poly(G/I)-tailed RNA product was subjected to RT-PCR and the reaction product was loaded onto a 2.5% agarose TBE gel.

### Nuclear isolation and nuclear run-on analyses

MEFs lysates were resuspended in nuclear extraction buffer [10 mM Tris-HCl pH 7.5, 2 mM MgCl_2_, 3 mM CaCl_2_, 250 units of SUPERaseIn, and 10% glycerol] and incubated on ice for 10 min. Lysates were then mechanically lysed by passing the suspension 10 times through a 26G needle. Nuclei were pelleted by centrifugation and resuspended in 1 ml of nuclear extraction buffer followed by gentle pipetting up and down 10 times. The nuclei were centrifuged and resuspended in 100 μL of storage solution (50 mM Tris-Cl pH 8, 5 mM MgCl_2_, 0.1 mM EDTA, 40% glycerol, and 50 units of SUPERaseIn). For nuclear run-on analyses, nuclei were incubated with 300 μL of nuclear run-on reaction buffer [(10 mM Tris-Cl pH 8.0, 5 mM MgCl_2_, 1 mM DTT, 300 mM KCl and 200 units of SUPERaseIn, 1% sarkosyl, 4 mM ATP, 1 mM CTP, 1 mM GTP and 200 mM EU]. The reaction mixtures were incubated at 37 °C for 30 min followed by nuclear RNA isolation.

### Nascent RNA purification and cDNA preparation

Nascent RNAs were purified from total nuclear RNA samples using a Click-iT Nascent RNA Capture Kit according to the manufacturer's instructions. Briefly, biotin-azide was attached to ethylene-groups of EU-labeled RNA using click-it chemistry. EU-labeled nascent RNA was purified using Streptavidin magnetic Dynabeads. The preparation of cDNA was performed using nascent RNA captured on the beads. The cDNA synthesis reaction mix [6 μg random hexamer, 2 μg oligo dT, 10 mM dNTP mix and 14 μl buffer from the Click-iT Nascent RNA Capture Kit in a total of 20 μl] was added to these beads and incubated at 70 °C for 10 min. Next, 4 μl 10 x RT buffer, 20 mM MgCl_2_, 0.1 M DTT, 20 U/μl SuperaseIn, and 200 U/μl SuperScript III Reverse Transcriptase were added to the mixture and incubated at 25 °C for 10 min, 50 °C for 50 min, and 85 °C for 5 min for first-strand cDNA synthesis (Table S9).

### ChIP analyses

For ChIP analysis, MEFs and hepatocytes were crosslinked with 1% formaldehyde at room temperature for 20 min. The reaction was halted with 125 mM glycine. These cells were washed twice in PBS and collected by centrifugation. The nuclei were isolated by resuspending the cells in cell lysis buffer (25 mM HEPES pH 8.0, 10 mM KCl, 0.1% NP40, 1.5 mM MgCl_2_, 1 mM DTT, and a protease inhibitor cocktail) at 4 °C for 20 min. Isolated nuclei were re-suspended in nuclei lysis buffer (50 mM HEPES pH 8.0, 140 mM NaCl, 1 mM EDTA, 1% Triton X-100, 0.1% sodium deoxycholate, 0.1% SDS, and a protease inhibitor cocktail) and sonicated. The samples were immunoprecipitated with the appropriate antibodies in combination with Protein-A-Sepharose beads. These precipitates were extracted with elution buffer (10 mM Tris-HCl, pH 8.1, 1% SDS, and 1 mM EDTA). The eluted samples were pooled and heated at 65 °C overnight to reverse the formaldehyde cross-linking. The DNA fragments were purified with a QIAquick spin kit. The qPCR reactions were performed with SYBR Green PCR Master Mix in a MicroAmp optical reaction plate using QuantStudio 3. Primers used for qPCR are summarized in Table S5.

### ChIP-Seq library preparation, alignment and data analyses

To validate that the anti-Ssu72 antibody (Cell Signaling) is feasible for ChIP-seq, we performed Western blotting after immunoprecipitating the formaldehyde fixed cells using the anti-Ssu72 antibody. We observed the pull-down of endogenous Ssu72 in Ad-Luc- and Ad-Cre-infected MEFs, indicating that this antibody is able to pull down crosslinked endogenous Ssu72 protein. Then, 30 to 50 million MEFs, hepatocytes and ES cells were crosslinked with 1% paraformaldehyde in PBS for 10 min at RT and were quenched with 0.2 M glycine for 5 min. For sonication of cells fixed for 10 min, the optimal sonication conditions were generated a DNA smear with more than about 90% of total DNA fragments less than 1 kb. To normalize the ChIP-seq signal, we employed a ChIP-seq spike in, using Drosophila chromatin (Active Motif) according to the manufacturer's instructions, for the Ssu72, Pol II and phospho-Pol II ChIP-Seq analyses. The ChIP-seq DNA libraries were multiplexed and sequenced using a TruSeq ChIP Samples Prep Kit on an Illumina NextSeq machine. A total of 50,000,000-103,000,000 unique reads were generated per sample. To generate the ChIP-seq data, ChIP enriched DNA reads were mapped to UCSC mouse genome (mm10) using BWA. All duplicate tags with identical sequences and possible sequencing artifacts were filtered out with the PICRAD 1.76 pipeline. The ChIP-Seq read density values were normalized per million mapped reads. The peaks of ChIP-Seq that overlapped with the possible anomalous artifact regions (such as high-mapping regions or satellite repeats) blacklisted by the ENCODE consortium (https://sites.google.com/site/anshulkundaje/projects/blacklists) were removed using BEDTools. To generate average occupancy plots against ChIP-Seq results of Ssu72, Pol II and phosho-Pol II in the MEFs and hepatocytes, we selected all genes with a distance of at least 4kb from any other known annotation (mRNAs, snRNAs, snoRNAs, tRNAs) by using mouse mm10 Refseq gene annotations. Based on selected genes, we retrieved the occupancy values from - 4 kb upstream of TSS to + 4 kb downstream of TES for total genes selected with a distance of at least 4 kb. Mapped reads were extended to 150 bp toward the interior of the sequenced fragment and input normalized to total reads aligned. The average coverage was binned in 20-bp intervals. Peak calling was performed using MACS 1.4.2 to determine statistical enrichment at a stringent p-value (p < 1E-5 ~p < 1E-7). Peaks within 1000 bp to the nearest TSS were set as promoter. The distribution of peaks (as intronic, intergenic, exonic, intronic etc.) was annotated using findMotifsGenome.pl. Metagene plots of ChIP-seq were performed using NGSplot. Density plots were generated by counting tags using indicated window size with 20 bp bin. Each plot was involved in 4 kb upstream of TSS, the gene body, and 4 kb downstream of the TES. Different occupancy-profiling categories (high 0~30%, intermediate 30~50%, and low 50~100%) were calculated and classified based on the average ChIP signal from 4kb upstream of the TSS to 4kb downstream of the 3' end of all selected genes for each experiment. For example, 515 of Ssu72-bound genes were selected in MEFs, 1994 in hepatocytes and 680 in ES cells through ChIP-Seq results. The 515 genes selected in MEFs were subdivided into groups of 155, 103 and 257 based on the average ChIP signal; 1994 genes selected in hepatocytes were subdivided into groups of 599, 398 and 997; 680 genes selected in ES cells were subdivided into 204, 136 and 340, as represented by differentially colored lines. Heatmaps of signal intensity of the ChIP samples were generated using deepTools. In brief, computeMatrix was used to calculate the signal intensity scores per ChIP sample in a given genome region that was specified by bed files. The output of computeMatrix was a matrix file of scores. The heatmap generated using plotHeatmap.

### Data access

All raw and processed sequencing data that support the findings of this study have been deposited in Gene Expression Omnibus (GEO). The accession number is GSE122101.

### Quantification and statistical analysis

Data are presented as mean ± SD. Statistical significance was tested with Student's t-test for two groups or one-way ANOVA for multiple groups with similar size. When exact p values are not indicated, they are represented as follows: ^∗^, p < 0.05; ^∗∗^, p < 0.01; ^∗∗∗^, p < 0.001; n.s., p > 0.05.

## Supplementary Material

Supplementary figures and methods.

Supplementary table 1. List of down- and up-regulated genes by Ssu72 depletion in MEFs, hepatocytes, and ES cells.

Supplementary table 2. Ssu72 mainly controls the expression of actively transcribed gene groups with cell-type-specificity.

Supplementary table 3. Gene set enrichment analysis (GSEA) by Ssu72 depletion in MEFs, hepatocytes, and ES cells.

Supplementary table 4. Gene ontology results of RNA-Seq and Chip-Seq analyses.

Supplementary table 5. The peaks and target genes of Ssu72 ChIP-Seq data in MEFs, hepatocytes, and ES cells.

Supplementary table 6. Ssu72 binding proteomes.

Supplementary table 7. The primers for mouse genotyping.

Supplementary table 8. List of used antibody.

Supplementary table 9. The primers for qRT-PCR and ChIP analyses.

## Abbreviations

Ad-Cre: Cre recombinase adenovirus
Ad-Luc: luciferase adenovirus
Alb-Cre: albumin-driven Cre recombinase
BrdU: 5-bromo-2'-deoxyuridine
cDNA: complementary DNA
ChIP: chromatin immunoprecipitation
ChIP-qPCR: chromatin immunoprecipitation analysis coupled with qPCR
ChIP-Seq: chromatin immunoprecipitation coupled with high-throughput sequencing
CPF/CPSF: cleavage and polyadenylation factor
CPM: counts per million
CTD: C-terminal domain
DSIF: DRB sensitivity-inducing factor
DTT: dithiothreitol
EGF: epidermal growth factor
ES: embryonic stem
EU: ethylene uridine
Fcp1: TFIIF-associated CTD phosphatase 1
FDR: false discovery rate
GO: Gene Ontology
GSEA: gene set enrichment analyses
GST: glutathione *S*-transferase
HA: hemagglutinin
Hexim1: HMBA-inducible protein 1
IEF: isoelectric focusing
Larp7: La-related protein 7
MEF: mouse embryonic fibroblast
ncRNA: non-coding RNA
NELF: negative elongation factor
Pin1: peptidyl-prolyl *cis*/*trans* isomerase
PMSF: phenylmethylsulfonyl fluoride
Pol II: RNA polymerase II
Pold2: DNA polymerase delta subunit 2
PNUTS: PP1 nuclear targeting subunit
PNUTS-PP1: PP1 nuclear targeting subunit-serine/threonine protein phosphatase type 1
PP2A: serine/threonine protein phosphatase type 2A
PTD: protein transduction domain
P-TEFb: positive transcription elongation factor b
RNA-Seq: RNA sequencing
RPAP2: RNA polymerase II-associated protein 2
snoRNA: small nucleolar RNA
snRNA: small nuclear RNA
snRNP: small nuclear ribonucleoprotein
TAP: tandem affinity purification
TES: transcription end site
TR: traveling ratio
TSS: transcription start site
