# Supplementary material for: Mammalian Ssu72 phosphatase preferentially considers tissue-specific actively transcribed gene expression by regulating RNA Pol II transcription
Source: Theranostics. 2022 Jan 1;12(1):186–206. doi: 10.7150/thno.62274 (PMC8690912; doi:10.7150/thno.62274)
Supplement: Supplementary file 1 — Supplementary figures and methods. [file thnov12p0186s1.pdf]

Supplementary Material

Mammalian Ssu72 phosphatase preferentially considers tissue-specific actively transcribed gene expression by regulating RNA Pol II transcription

(Running title: Tissue-specific plasticity regulated by Ssu72)

Hyun-Soo Kim[1,✉], Yoon Jeon[2], Yoon Ok Jang[3], Ho Lee[2], Yong Shin[3,✉], Chang-Woo Lee[1,✉]

[1]Department of Molecular Cell Biology, Sungkyunkwan University School of Medicine, Suwon 16419, South Korea

[2]Graduate School of Cancer Science and Policy, Research Institute, National Cancer Center, Goyang 10408, South Korea

[3]Department of Biotechnology, College of Life Science and Biotechnology, Yonsei University, Seoul 03722, South Korea

✉Corresponding authors: Yong Shin (shinyongno1@yonsei.ac.kr; Tel.: +82-2-2123-2885); Hyun-Soo Kim (jazz7780@skku.edu; Tel.: +82-2-2123-2885); Chang-Woo Lee (cwlee1234@skku.edu; Tel.: +82-31-299-6121)

This file includes:

Figures S1 to S12

Supplementary methods

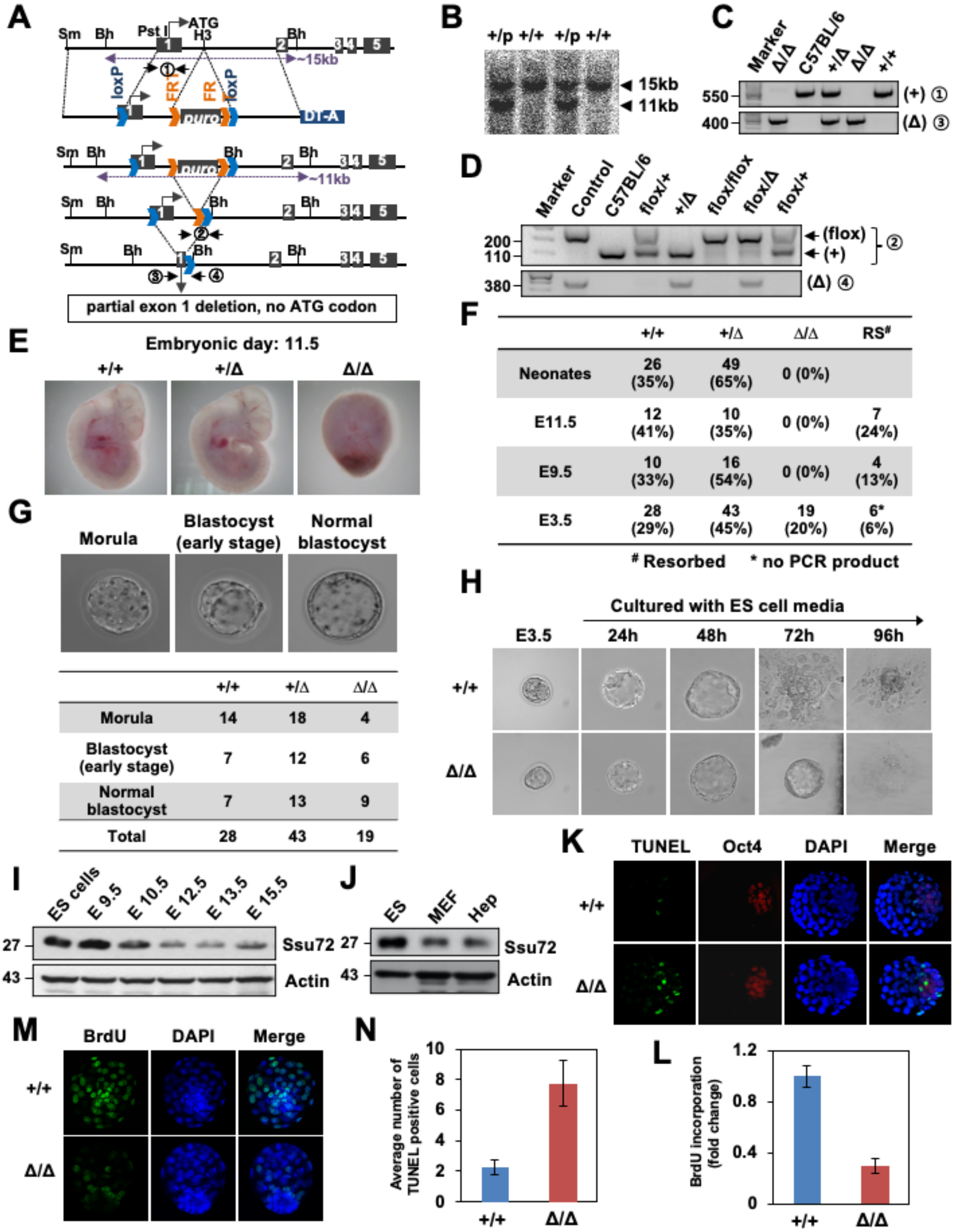
A
Sm Bh Pst I ATG H3 Bh
~15kb
loxP FR FR loxP
puro DT-A
~11kb
partial exon 1 deletion, no ATG codon
B
+/p +/+ +/p +/+
15kb
11kb
C
Marker Δ/Δ C57BL/6 +/Δ Δ/Δ +/+
550 (+) ①
400 (Δ) ③
D
Marker Control C57BL/6 flox/+ +/Δ flox/flox flox/Δ flox/+
200 (flox)
110 (+) ②
380 (Δ) ④
E
Embryonic day: 11.5
+/+ +/Δ Δ/Δ
F
+/+ +/Δ Δ/Δ RS#
Neonates 26 (35%) 49 (65%) 0 (0%)
E11.5 12 (41%) 10 (35%) 0 (0%) 7 (24%)
E9.5 10 (33%) 16 (54%) 0 (0%) 4 (13%)
E3.5 28 (29%) 43 (45%) 19 (20%) 6* (6%)
Resorbed * no PCR product
G
Morula Blastocyst (early stage) Normal blastocyst
+/+ +/Δ Δ/Δ
Morula 14 18 4
Blastocyst (early stage) 7 12 6
Normal blastocyst 7 13 9
Total 28 43 19
H
Cultured with ES cell media
E3.5 24h 48h 72h 96h
+/+
Δ/Δ
I
ES cells E 9.5 E 10.5 E 12.5 E 13.5 E 15.5
27 Ssu72
43 Actin
J
ES MEF Hep
27 Ssu72
43 Actin
K
TUNEL Oct4 DAPI Merge
+/+
Δ/Δ
M
BrdU DAPI Merge
+/+
Δ/Δ
N
Average number of TUNEL positive cells
0 2 4 6 8 10
+/+ Δ/Δ
L
BrdU incorporation (fold change)
0 0.4 0.8 1.2
+/+ Δ/Δ

Figure S1. Ssu72 loss causes developmental defects in mouse embryos.

(A) Schematic representation of the Ssu72 locus, the targeting vector, and targeted, floxed, and deleted loci. Exon 1–5, translation start site (ATG), puromycin cassette (puro), diphtheria toxin A chain gene (DT-A), and restriction sites (Sm, *Sma*I; Bh, *Bam*HI; P, *Pst*I; H3, *Hind*III; Kp, *Kp*nI) are shown in the Ssu72 locus. The murine Ssu72 gene located on chromosome 4E2 is composed of five exons with a potential translational start codon in exon 1 (NCBI annotation of NCBI Build 37.2). The targeting strategy was designed to flank Ssu72 exon 1 with *loxP* sites, allowing Cre-mediated exon 1 deletion. (B) $Ssu72^{+/+}$ and $Ssu72^{+/p}$ ES cell clones were subjected to Southern blot analyses. After *Bam*HI digestion, bands representing wild-type and mutant alleles are ~15 and ~11 kb, respectively. The symbol "p" represents the puromycin allele. (C) PCR analyses with genomic DNA extracted from mouse tails. The circled number represents a PCR reaction with primers described in Figure S1A. (D) PCR analyses with genomic DNA from mice containing floxed and deleted loci. The circled number represents a PCR reaction with primers described in Figure S1A. (E) Images of wild-type ($^{+/+}$), heterozygous ($^{+/\Delta}$), and homozygous ($^{\Delta/\Delta}$) mutant embryos at embryonic day 11.5 (E11.5). Heterozygous $Ssu72^{+/\Delta}$ mice were seemingly normal, healthy, and fertile without developmental abnormalities during a 12-month or longer observation period. (F) Genotypes of progeny from Ssu72-heterozygous intercrosses by PCR analysis are shown. (G) Images of embryos at embryonic day 3.5 (E3.5). Those embryos were classified according to their appearance: morula, blastocyst (early stage), and normal blastocyst. Genotypes of embryos at E3.5 from Ssu72-heterozygous intercrosses are shown in the table. Genotyping analysis of 90 embryos from $Ssu72^{+/\Delta}$ mice intercrosses at embryonic days 3.5–4.5 (E3.5–4.5) revealed that 28 embryos were

wild-type ($^{+/+}$), 43 embryos were $^{+/\Delta}$, and 19 embryos were $^{\Delta/\Delta}$. (H) Blastocyst stage embryos recovered at E3.5 were cultured *in vitro* for 24, 48, 72, and 96 h. (I) Expression levels of Ssu72 in indicated mouse ES cells during early embryogenesis. (J) Expression levels of Ssu72 in ES cells, MEFs, and hepatocytes. (K) Ssu72$^{+/+}$ and Ssu72$^{\Delta/\Delta}$ embryos were fixed and double-stained for TUNEL (green) and Alexa 546 (Oct4; red). (L) Graph representing the average number of TUNEL-positive cells. Error bars indicate SD. (M) BrdU-incorporated Ssu72$^{+/+}$ and Ssu72$^{\Delta/\Delta}$ embryos were fixed and immunostained with anti-BrdU (green). (N) Graph representing values of relative BrdU incorporation. Error bars indicate SD.

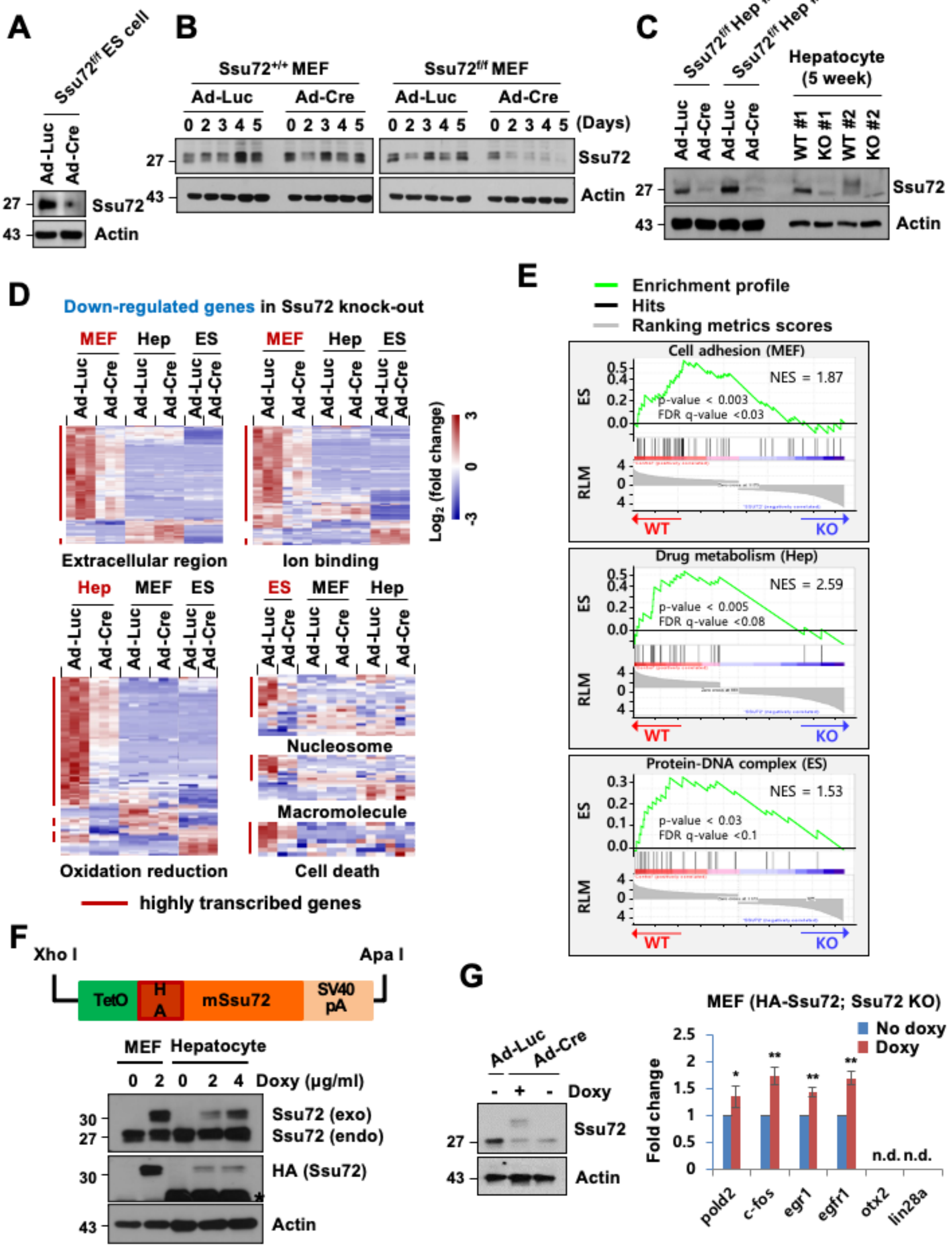
A
Ssu72f/f ES cell
Ad-Luc
Ad-Cre
27
43
Ssu72
Actin
B
Ssu72+/+ MEF
Ssu72f/f MEF
Ad-Luc
Ad-Cre
0 2 3 4 5
(Days)
Ssu72
Actin
C
Ssu72f/f Hep #1
Ssu72f/f Hep #2
Hepatocyte
(5 week)
WT #1
KO #1
WT #2
KO #2
D
Down-regulated genes in Ssu72 knock-out
MEF
Hep
ES
Extracellular region
Ion binding
Log2 (fold change)
Oxidation reduction
Nucleosome
Macromolecule
Cell death
highly transcribed genes
E
Enrichment profile
Hits
Ranking metrics scores
Cell adhesion (MEF)
NES = 1.87
p-value < 0.003
FDR q-value <0.03
Drug metabolism (Hep)
NES = 2.59
p-value < 0.005
FDR q-value <0.08
Protein-DNA complex (ES)
NES = 1.53
p-value < 0.03
FDR q-value <0.1
ES
RLM
WT
KO
F
Xho I
Apa I
TetO
HA
mSsu72
SV40 pA
MEF
Hepatocyte
Doxy (μg/ml)
Ssu72 (exo)
Ssu72 (endo)
HA (Ssu72)
Actin
G
Ad-Luc
Ad-Cre
Doxy
Ssu72
Actin
MEF (HA-Ssu72; Ssu72 KO)
No doxy
Doxy
Fold change
n.d. n.d.
pold2
c-fos
egr1
egfr1
otx2
lin28a

Figure S2. Validations of Cre adenoviruses, RNA-Seq heatmap data, GSEA analyses, and HA-Ssu72 transgenic mice.

(A) Ssu72$^{f/f}$ ES cells were infected with Ad-Luc and Ad-Cre for 12 h and cultured for 3 days with fresh media. ES cells were harvested and immunoblotted with anti-Ssu72 and anti-actin antibodies. (B) Ssu72$^{+/+}$ and Ssu72$^{f/f}$ MEFs were infected with Ad-Luc and Ad-Cre for 12 h. At the indicated time, MEF cells were harvested and immunoblotted with anti-Ssu72 and anti-actin antibodies. (C) Ad-Luc-infected or Ad-Cre-infected hepatocyte extracts were immunoblotted with antibodies indicated (left panels). Hepatocytes from Ssu72$^{f/f}$ mice and Alb-Cre;Ssu72$^{f/f}$ mice were harvested and immunoblotted with antibodies indicated (right panels). (D) Gene ontology-based heatmaps of down-regulated genes between control (Ad-Luc-infected) and Ssu72-depleted (Ad-Cre-infected) MEFs, hepatocytes, and ES cells. Subsets of genes down-regulated by Ssu72 depletion in each cell type (red) are compared with different cell types (black). Red lines denote transcriptionally active genes in each cell type. The color bar represents the gradient of log2-fold changes for each cell type. (E) Gene set enrichment analysis (GSEA) enrichment plot of down-regulated gene sets in Ssu72-depleted MEFs, hepatocytes, and ES cells versus related gene sets (MEF: cell adhesion; Hep: drug metabolism; ES: protein–DNA complex). The green curve shows the enrichment score, reflecting the degree to which each gene was enriched (black vertical lines). (F) The vector construct used to generate doxycycline (Doxy)-inducible HA-tagged Ssu72 transgenic mice. TetO (promoter containing tetracycline operator sequence), HA (hemagglutinin epitope tag), mSsu72 (mouse Ssu72 sequence), and SV40pA (SV40 early polyadenylation signal) are shown. The vector was linearized, purified, and injected into the pronuclei of fertilized C57BL/6J mice. MEFs and

hepatocytes expressing HA-tagged Ssu72 were cultured in the absence (−) or presence (+) of Doxy. Cells were harvested and subsequently immunoblotted with indicated antibodies. (G) MEF extracts of Ssu72 WT, Ssu72 KO, and Ssu72 KO; HA-Ssu72 mice were immunoblotted with indicated antibodies. qPCR analyses of expression levels of various genes using Doxy-inducible HA-tagged Ssu72 transgenic MEFs under the Ssu72-depleted condition. Data are representatives of three independent experiments. Error bars indicate SD.

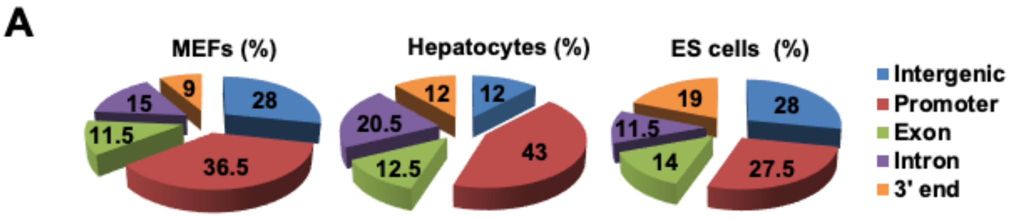

Distribution	MEFs_Ssu72 peaks	Hepatocytes_Ssu72 peaks	ES cells_Ssu72 peaks
Intergenic	1396	1349	1125
Promoter	1823	4835	1107
Exon	573	1404	563
Intron	748	2303	462
3' end	449	1346	763
Total	4989	11237	4020

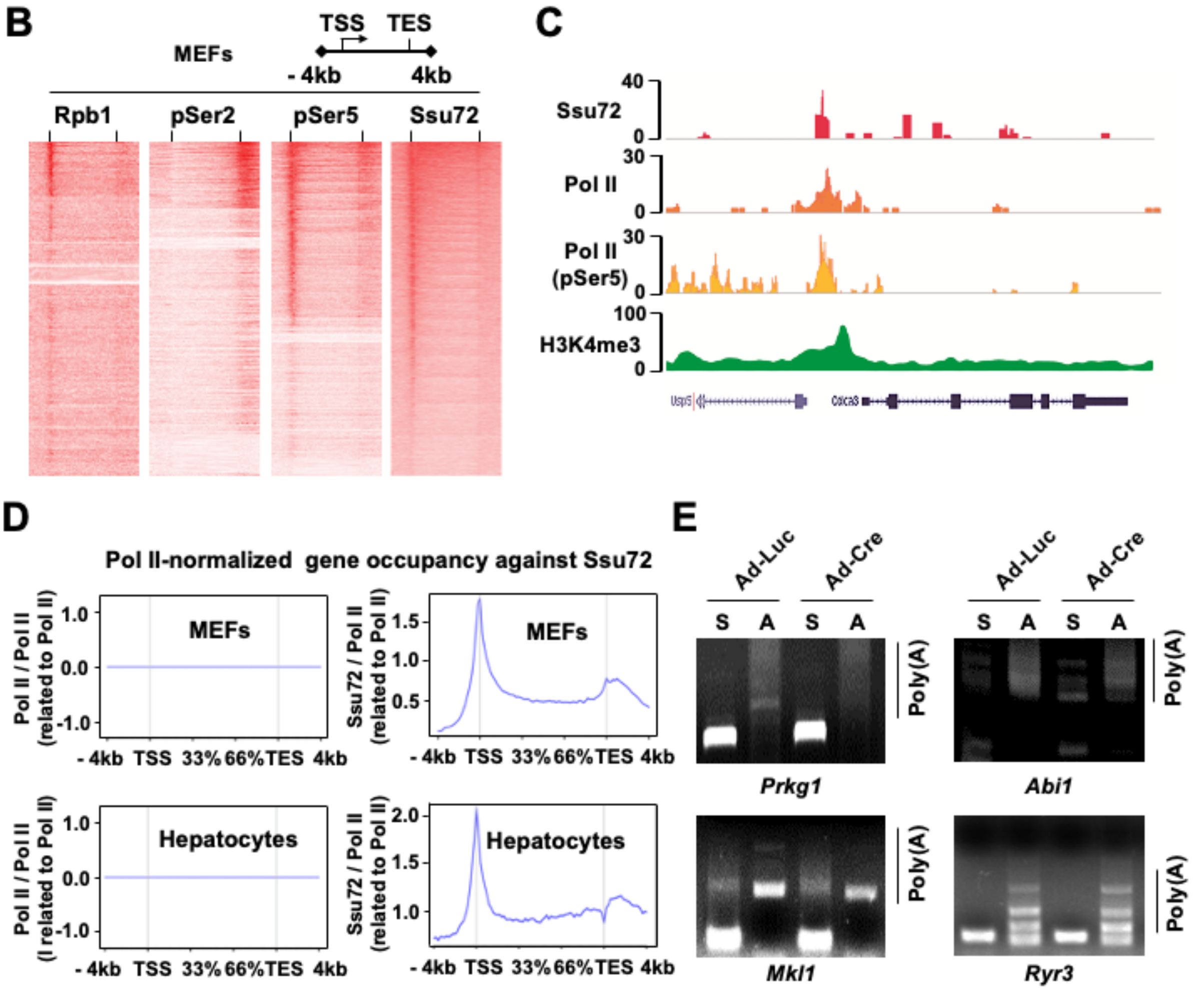

Figure S3. Distribution of enriched Ssu72 ChIP-Seq signals.

(A) Pie chart and table showing the genomic distribution of Ssu72 ChIP-Seq peaks with respect to various gene landmarks, including promoter (within ± 1 kb of TSS), exon, intron, 3′-end (within 4 kb downstream of the gene), and intergenic region in MEFs, hepatocytes, and ES cells. (B) Density plots of Rpb1 (first column), pSer2 (second column), pSer5 (third column) and Ssu72 (fourth column) distributions at individual genes in MEFs. Each panel represents 4 kb upstream of TSS, the gene body, and 4 kb downstream of the TES. (C) ChIP-Seq signals for Ssu72, Pol II, pSer5, and H3K4me3 (GSE26657) across the indicated loci. (D) Pol II-normalized average gene occupancies of Ssu72 ChIP-Seq signals in MEFs and hepatocytes. (E) Comparison of mouse poly(A) tail-lengths in MEFs. S: gene specific PCR primer; A: Poly(A) tail PCR primer.

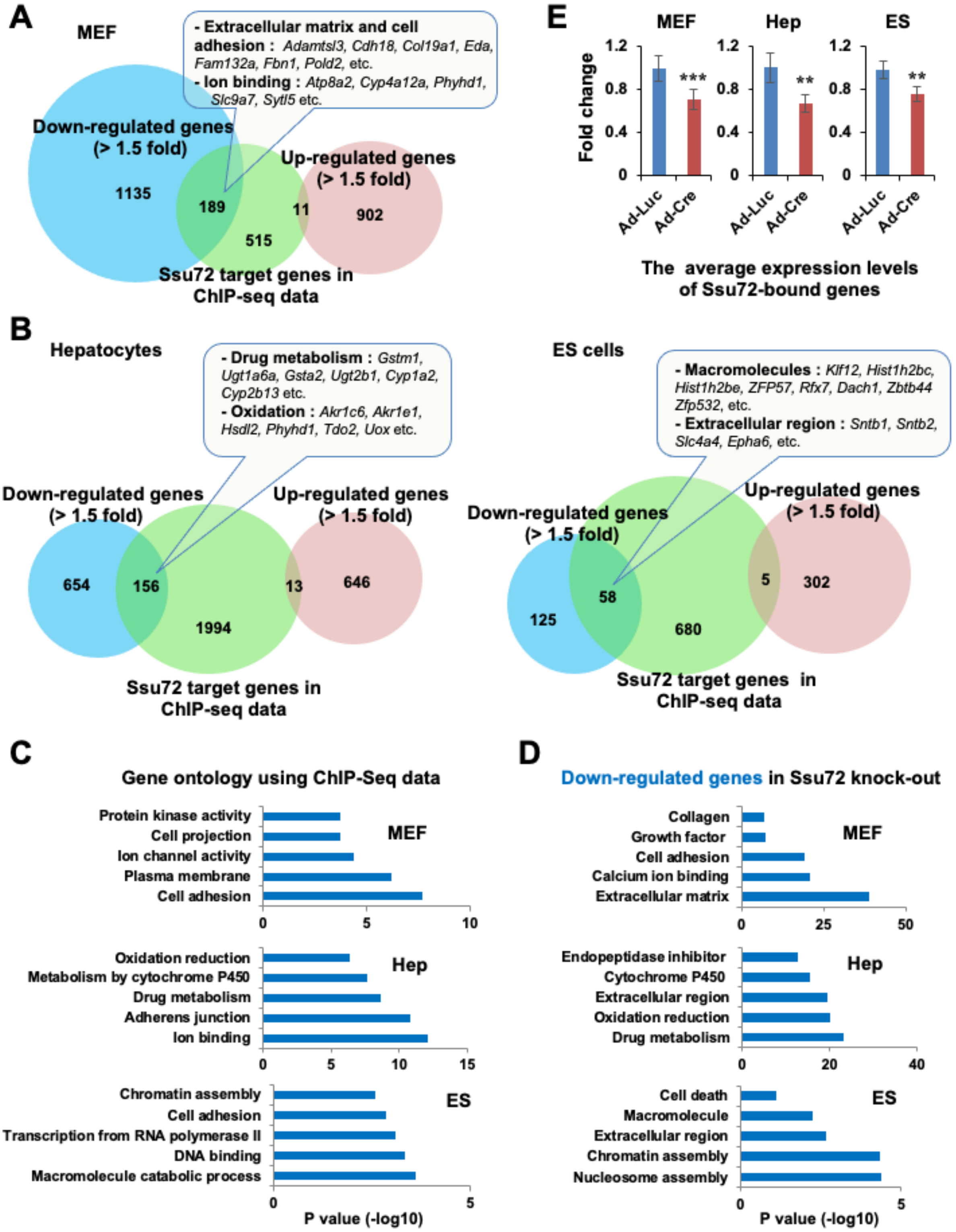

A
MEF
- Extracellular matrix and cell adhesion : Adamtsl3, Cdh18, Col19a1, Eda, Fam132a, Fbn1, Pold2, etc.
- Ion binding : Atp8a2, Cyp4a12a, Phyhd1, Slc9a7, Sytl5 etc.
Down-regulated genes (> 1.5 fold)
Up-regulated genes (> 1.5 fold)
1135
189
11
902
515
Ssu72 target genes in ChIP-seq data
E
MEF
Hep
ES
Fold change
1.2
0.8
0.4
0
***
**
**
Ad-Luc
Ad-Cre
The average expression levels of Ssu72-bound genes
B
Hepatocytes
- Drug metabolism : Gstm1, Ugt1a6a, Gsta2, Ugt2b1, Cyp1a2, Cyp2b13 etc.
- Oxidation : Akr1c6, Akr1e1, Hsdl2, Phyhd1, Tdo2, Uox etc.
Down-regulated genes (> 1.5 fold)
Up-regulated genes (> 1.5 fold)
654
156
13
646
1994
Ssu72 target genes in ChIP-seq data
ES cells
- Macromolecules : Klf12, Hist1h2bc, Hist1h2be, ZFP57, Rfx7, Dach1, Zbtb44 Zfp532, etc.
- Extracellular region : Sntb1, Sntb2, Slc4a4, Epha6, etc.
Down-regulated genes (> 1.5 fold)
Up-regulated genes (> 1.5 fold)
125
58
5
302
680
Ssu72 target genes in ChIP-seq data
C
Gene ontology using ChIP-Seq data
MEF
Protein kinase activity
Cell projection
Ion channel activity
Plasma membrane
Cell adhesion
0 5 10
Hep
Oxidation reduction
Metabolism by cytochrome P450
Drug metabolism
Adherens junction
Ion binding
0 5 10 15
ES
Chromatin assembly
Cell adhesion
Transcription from RNA polymerase II
DNA binding
Macromolecule catabolic process
0 5
P value (-log10)
D
Down-regulated genes in Ssu72 knock-out
MEF
Collagen
Growth factor
Cell adhesion
Calcium ion binding
Extracellular matrix
0 25 50
Hep
Endopeptidase inhibitor
Cytochrome P450
Extracellular region
Oxidation reduction
Drug metabolism
0 20 40
ES
Cell death
Macromolecule
Extracellular region
Chromatin assembly
Nucleosome assembly
0 5
P value (-log10)

Figure S4. The validation analyses by integrating Ssu72-related ChIP-Seq and RNA-Seq data

(A and B) Venn diagram representing the overlap between the target genes against high-confidence Ssu72 ChIP-Seq peaks and the down- and up-regulated genes in Ssu72-depleted MEFs, hepatocytes and ES cells. (C) Gene ontology analyses of the Ssu72 target genes based on ChIP-Seq data of MEFs, hepatocytes, and ES cells. (D) Gene ontology analyses of the downregulated genes by Ssu72 depletion in MEFs, hepatocytes, and ES cells. (E) The average mRNA expression levels of the Ssu72-bound genes against high-confidence Ssu72 ChIP-Seq peaks in MEFs, hepatocytes, and ES cells based on RNA-Seq data. The asterisk denotes a difference according to Student's *t*-test (** $p < 0.01$, *** $p < 0.001$).

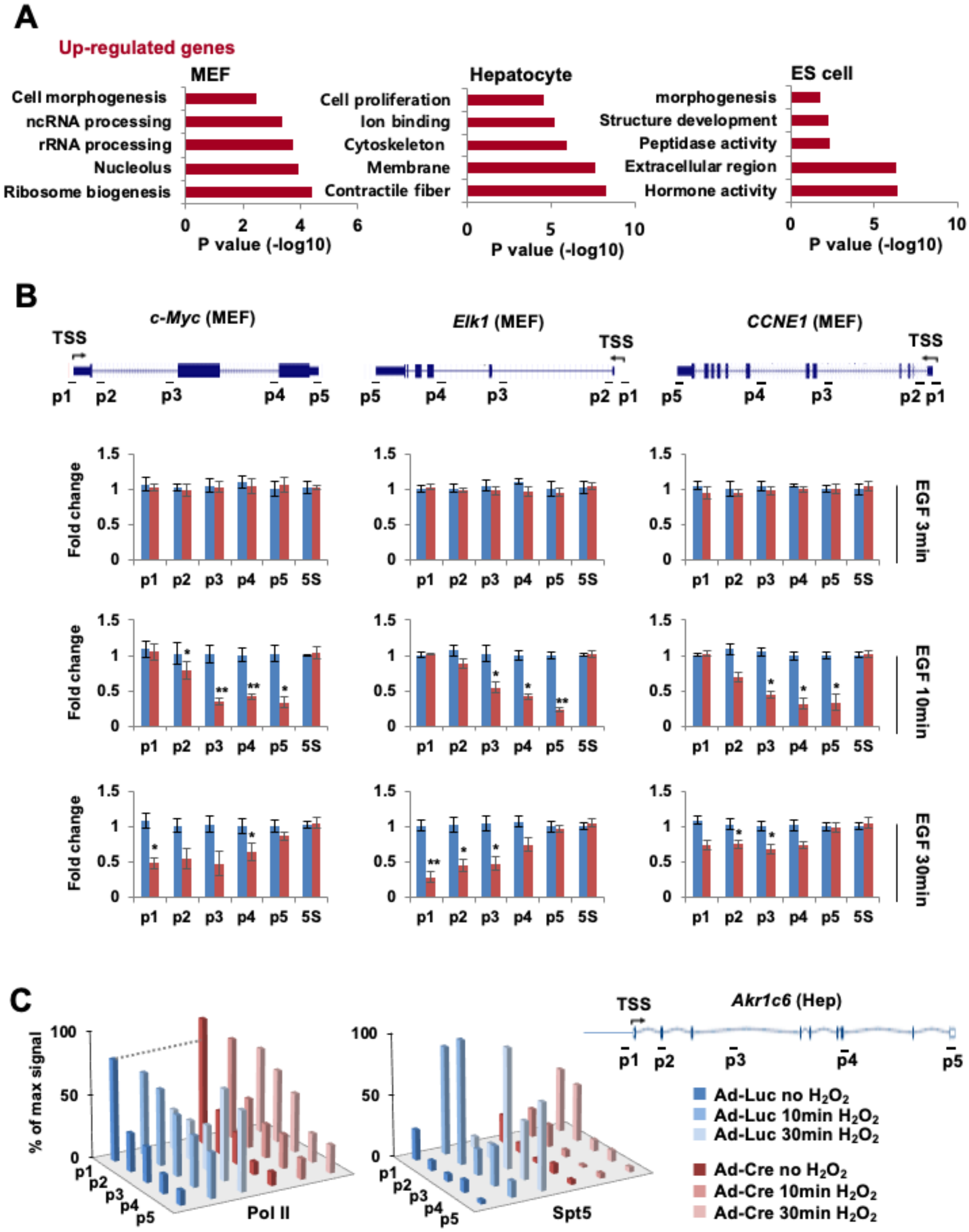

148

149

150

151

Figure S5. The characterization of upregulated genes caused by Ssu72 depletion, and ChIP analyses of Pol II and Spt5 in hepatocytes.

(A) Gene ontology analyses of the upregulated genes by Ssu72 depletion in MEFs, hepatocytes, and ES cells. (B) Serum-deprived Ad-Luc or Ad-Cre-infected MEFs treated with EGF for the indicated time periods and analyzed by qRT-PCR using intronic primers (p1 ~ p6) of indicated gene. Error bars indicate SD. (C) ChIP and qPCR analyses of Pol II and Spt5 using primers (p1 ~ p5) of Akr1c6 gene in H_2O_2 treated control and Ssu72-depleted hepatocytes. Prior to harvest, Ad-Luc or Ad-Cre-infected hepatocytes were stimulated with 100 μM H_2O_2 for 10 and 30 min, respectively. A schematic representation of Akr1c6 gene and locations of primer sets for qPCR analysis are shown in the upper panel.

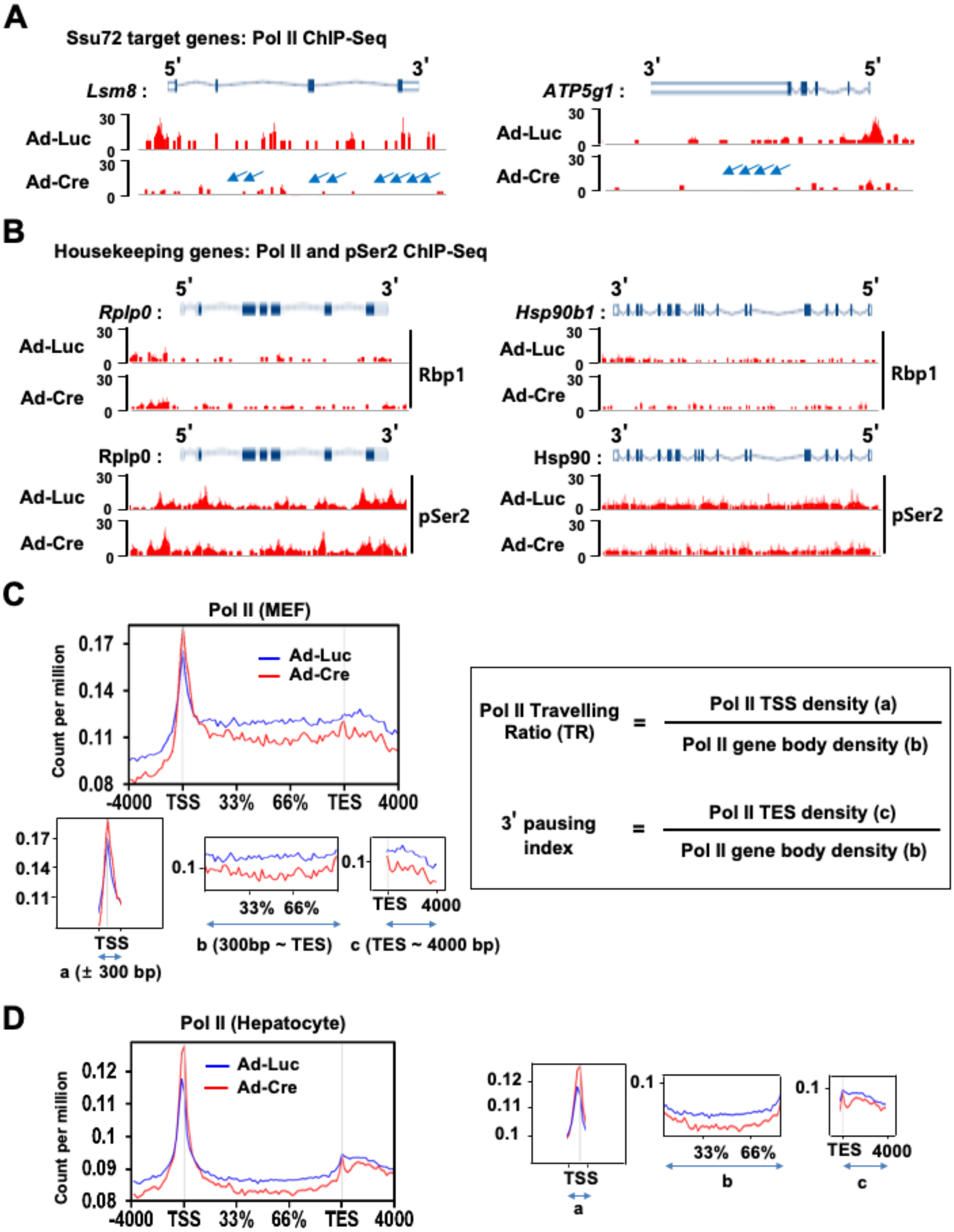
A
Ssu72 target genes: Pol II ChIP-Seq
Lsm8 :
ATP5g1 :
5'
3'
Ad-Luc
Ad-Cre
30
0
B
Housekeeping genes: Pol II and pSer2 ChIP-Seq
Rplp0 :
Hsp90b1 :
Hsp90 :
Rbp1
pSer2
C
Pol II (MEF)
Count per million
0.17
0.14
0.11
0.08
-4000
TSS
33%
66%
TES
4000
Pol II Travelling Ratio (TR)
=
Pol II TSS density (a)
Pol II gene body density (b)
3' pausing index
Pol II TES density (c)
0.1
a (± 300 bp)
b (300bp ~ TES)
c (TES ~ 4000 bp)
D
Pol II (Hepatocyte)
0.12
0.11
0.1
0.09
0.08
a
b
c

166

167

168

Figure S6. Genome browser views of Ssu72 target genes and housekeeping genes and traveling ratio calculation.

(A) Genome browser views of Pol II ChIP-Seq signals against various Ssu72 target genes in control and Ssu72-depleted MEFs. Schematic representation of several genes is shown at the top panel. Blue arrows indicate decreased Pol II signals in overall gene regions. (B) Genome browser views of Rpb1 (Pol II) and pSer2 ChIP-Seq signals against housekeeping genes (Rplp0 and Hsp90). (C) The genome-wide Pol II occupancy profiles describing the calculation used to determine the traveling ratio (TR) and 3' pausing index at each Pol II-bound gene in Ad-Luc and Ad-Cre infected MEFs. Promoter-proximal bin 'a' is defined using a fixed window from -300 to +300 bp around the annotated TSS. The gene body region 'b' bin is from + 300 bp to the annotated TES. The transcription termination region 'c' bin is from TES to + 4,000 bp after TES. TR is the ratio of Pol II density in the promoter-proximal 'a' bin to Pol II occupancy in the gene body region 'b' bin. The 3′ pausing index is ratio of Pol II density in the termination region 'c' bin to Pol II occupancy in the gene body region 'b' bin. (D) The TR and 3′ pausing index of Pol II occupancy profiles in Ad-Luc and Ad-Cre infected hepatocytes.

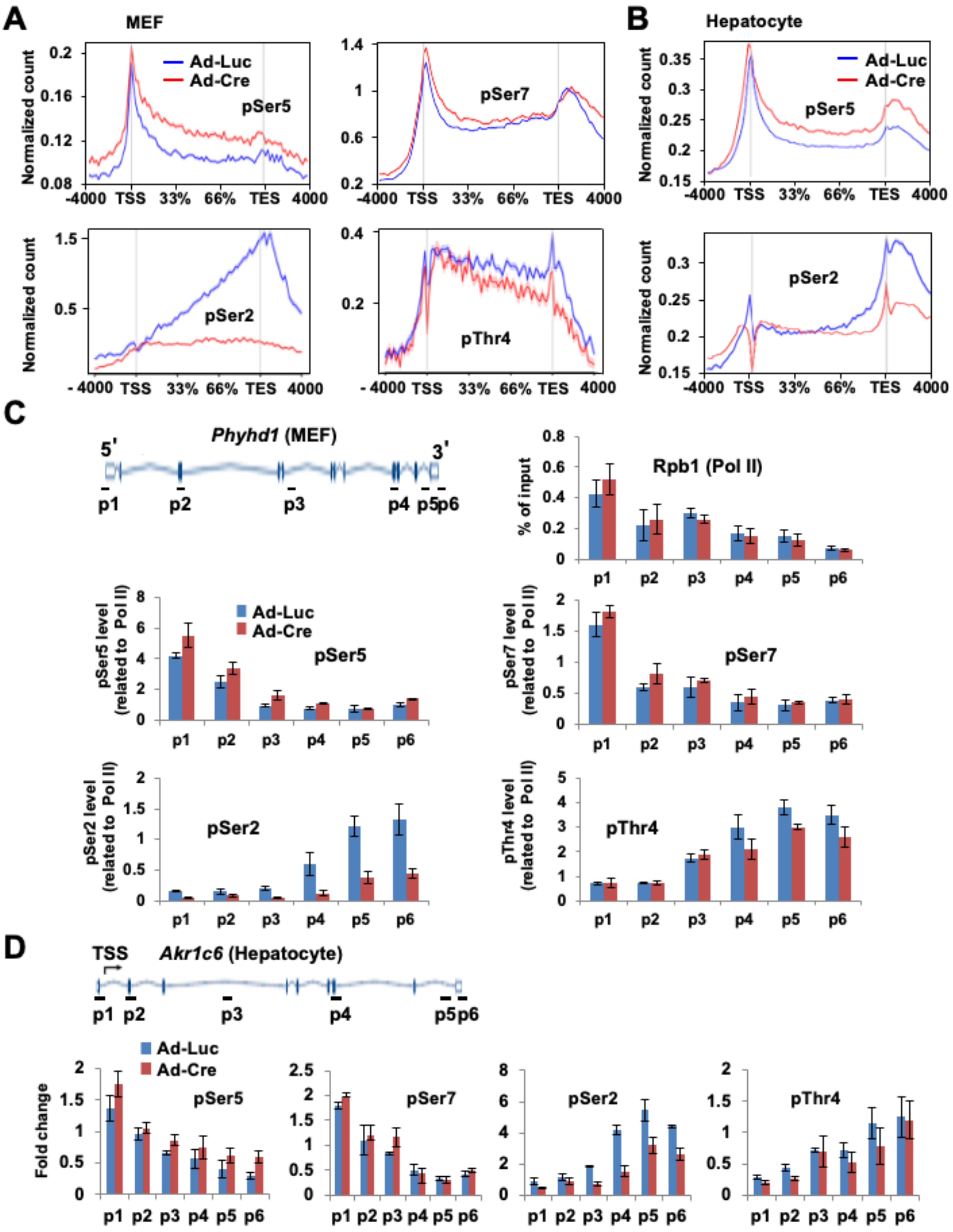
A
MEF
B
Hepatocyte
Ad-Luc
Ad-Cre
pSer5
pSer7
pSer2
pThr4
Normalized count
-4000 TSS 33% 66% TES 4000
C
Phyhd1 (MEF)
5'
3'
p1 p2 p3 p4 p5 p6
Rpb1 (Pol II)
% of input
pSer5 level (related to Pol II)
pSer7 level (related to Pol II)
pSer2 level (related to Pol II)
pThr4 level (related to Pol II)
D
TSS
Akr1c6 (Hepatocyte)
Fold change

Figure S7. Loss of Ssu72 results in abnormal phosphorylation of CTD code.

(A) Pol II-normalized average gene occupancies of pSer5-, pSer2-, pSer7-, and pThr4-Pol II phosphorylation in control (Ad-Luc) and Ssu72-depleted (Ad-Cre) MEFs. (B) Pol II-normalized average gene occupancies of pSer5- and pSer2-Pol II phosphorylation in control (Ad-Luc) and Ssu72- depleted (Ad-Cre) hepatocytes. (C) ChIP and qPCR analyses of Pol II and Pol II-normalized pSer5, pSer7, pSer2, and pThr4 against Phyhd1 gene in control and Ssu72-depleted MEFs. Data are represented as mean value ± SD of three independent experiments. (D) ChIP and qPCR analyses of pSer5, pSer7, pSer2, and pThr4 against Akr1c6 gene in control and Ssu72-depleted hepatocytes. Data are representatives of three independent experiments. Error bars indicate SD.

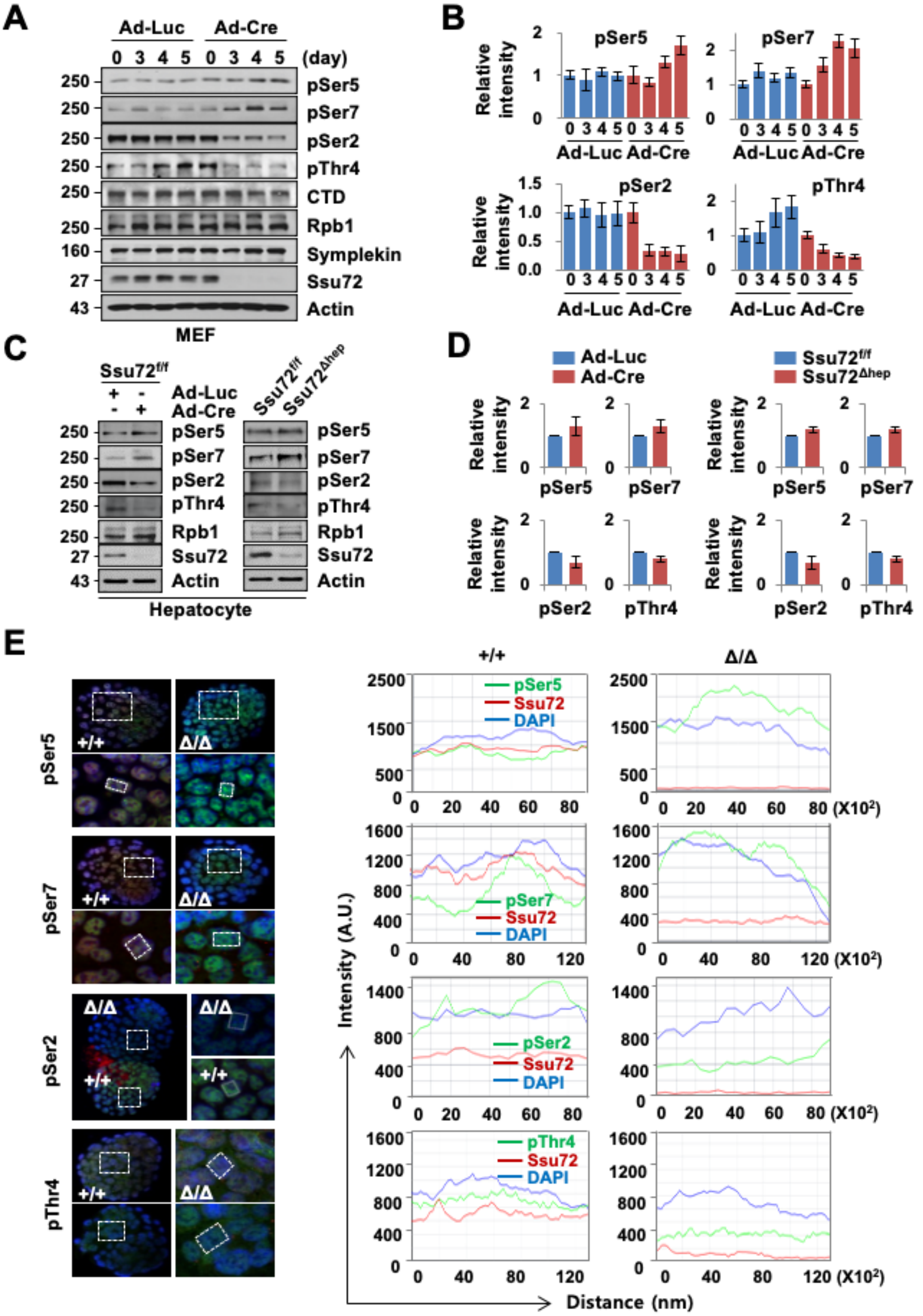
A
Ad-Luc
Ad-Cre
0 3 4 5 0 3 4 5 (day)
250 pSer5
250 pSer7
250 pSer2
250 pThr4
250 CTD
250 Rpb1
160 Symplekin
27 Ssu72
43 Actin
MEF
B
Relative intensity
pSer5
pSer7
pSer2
pThr4
Ad-Luc Ad-Cre
C
Ssu72f/f
Ssu72Δhep
Ad-Luc
Ad-Cre
pSer5
pSer7
pSer2
pThr4
Rpb1
Ssu72
Actin
Hepatocyte
D
Ad-Luc
Ad-Cre
Ssu72f/f
Ssu72Δhep
Relative intensity
pSer5
pSer7
pSer2
pThr4
E
+/+
Δ/Δ
pSer5
pSer7
pSer2
pThr4
Ssu72
DAPI
Intensity (A.U.)
Distance (nm)
(X10²)

Figure S8. Ssu72 is required for correct phosphorylation pattern of CTD in MEFs, hepatocytes and ES cells.

(A) Ad-Luc or Ad-Cre-infected MEF cell extracts immunoblotted with indicated antibodies. (B) Quantified band intensity of phosphorylated CTD residues shown in Figure S8A with ImageJ software. Data are representatives of five independent experiments. (C) Extracts of Ad-Luc or Ad-Cre-infected and albumin-Cre cross-mated hepatocytes analyzed by immunoblotting with indicated antibodies. (D) Quantified band intensity of phosphorylated CTD residues shown in Figure S8C. (E) Fixed Ssu72+/+ and Ssu72Δ/Δ embryos immunostained with anti-Ssu72 (Red) and various anti-phospho-CTD antibodies (Green; pSer5, pSer7, pSer2, and pThr4, respectively, Left panels). Representative single-cell traces (dotted square) of immunostained levels are quantified using ZEN 2012 blue edition software. Red, Ssu72; Green, pSer5, pSer7, pSer2, and pThr4; Blue, DAPI; Middle panels.

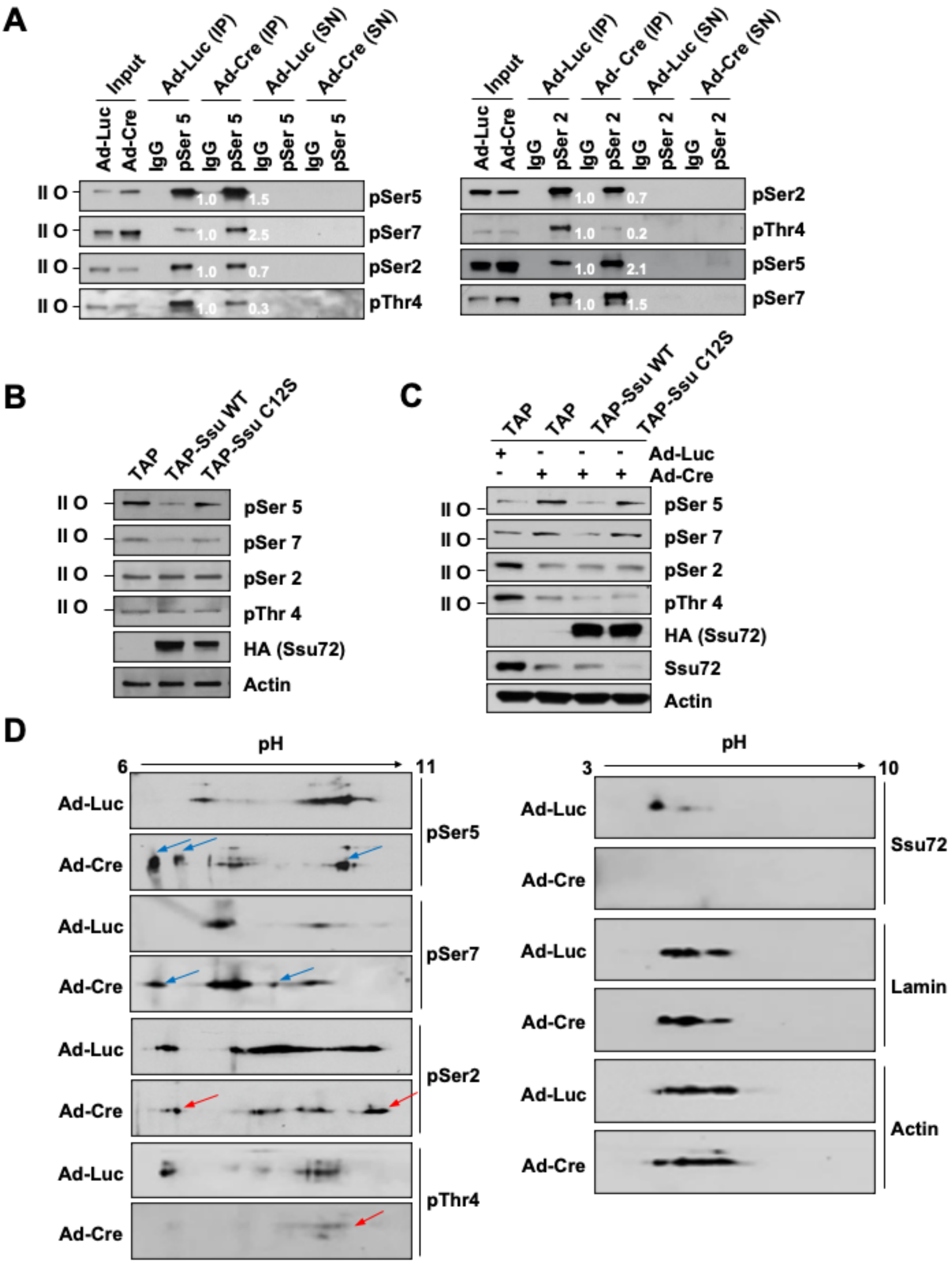
A
Input
Ad-Luc (IP)
Ad-Cre (IP)
Ad-Luc (SN)
Ad-Cre (SN)
Ad-Luc
Ad-Cre
IgG
pSer 5
II O
pSer5
pSer7
pSer2
pThr4
1.0
1.5
2.5
0.7
0.3
Ad- Cre (IP)
pSer 2
0.2
2.1
B
TAP
TAP-Ssu WT
TAP-Ssu C12S
pSer 5
pSer 7
pSer 2
pThr 4
HA (Ssu72)
Actin
C
Ssu72
D
pH
6
11
3
10
Lamin

Figure S9. The hyper-phosphorylation of Ser5 and Ser7 by Ssu72 depletion is directly coupled with the hypo-phosphorylation of Ser2 and Thr4

(A) Ad-Luc or Ad-Cre-infected MEF cell extracts were immunoprecipitated with normal IgG, pSer5, or pSer2 antibodies, and then separated into two fractions, the immuno-complex pellets (IP) and soluble supernatants (SN). Respective IP and SN samples were analyzed by immunoblotting with phospho-specific antibodies against pSer2, pSer5, pSer7, and pThr4. The signal intensity of each phospho-polypeptide was quantified using Image J software. (B) Ssu72$^{f/f}$ MEF cells were transfected with expression plasmids encoding TAP, TAP-Ssu72 WT, or TAP-Ssu72 C12S (phosphatase dead mutant). Cells were harvested and immunoblotted with the indicated antibodies. (C) Ssu72$^{f/f}$ MEF cells were transfected with TAP, TAP-Ssu72 WT, or TAP-Ssu72 C12S expression plasmids, and then infected with Ad-Luc or Ad-Cre. At 48 h post-transfection and further infection, cells were harvested and immunoblotted with the indicated antibodies. (D) Ad-Luc or Ad-Cre infected MEF cell extracts were analyzed by two-dimensional SDS-PAGE followed by immunoblotting with the indicated antibodies. Blue and red arrows indicate the acidic and basic shifts of phosphorylated CTD polypeptide spots, respectively.

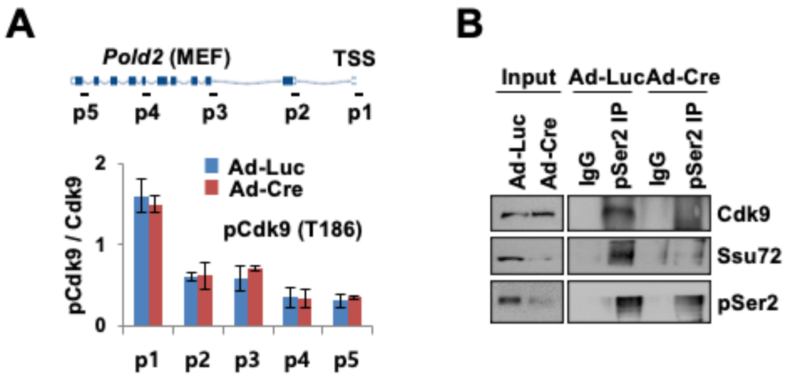

Figure S10. The interaction analyses of Pol II and Pol II pSer2 under control and Ssu72-depletion conditions

(A) ChIP analyses of total Cdk9-normalized phospho-Cdk9 (T186) against Pold2 gene in control and Ssu72-depleted MEFs. (B) Extracts from Ad-Luc or Ad-Cre-infected MEFs immunoprecipitated with IgG and anti-Pol II (pSer2) antibodies. Immunocomplexes were analyzed by Western blotting using indicated antibodies.

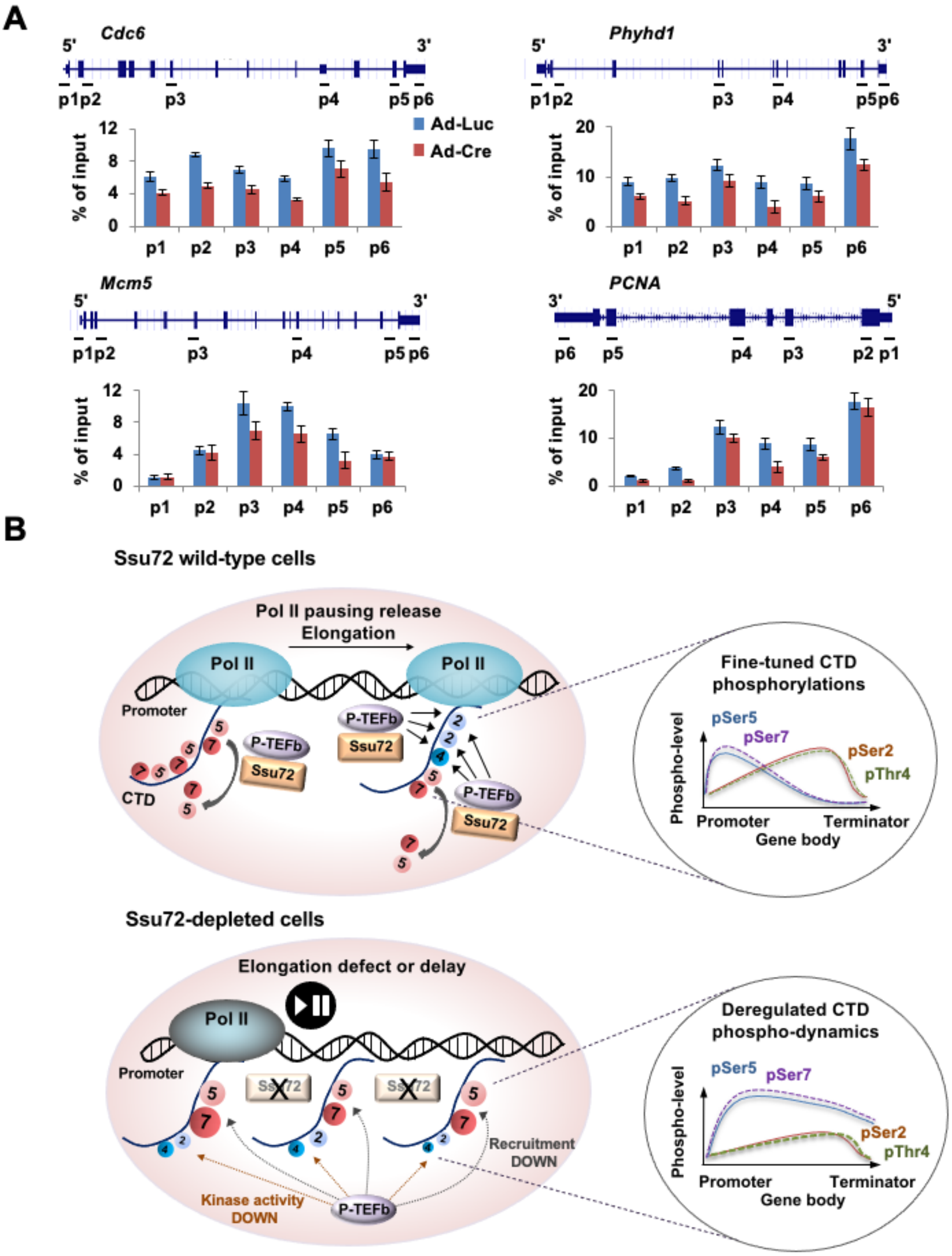
A
Cdc6
5'
3'
p1 p2
p3
p4
p5 p6
Ad-Luc
Ad-Cre
% of input
12
8
4
0
p1 p2 p3 p4 p5 p6
Phyhd1
5'
3'
p1 p2
p3
p4
p5 p6
% of input
20
10
0
p1 p2 p3 p4 p5 p6
Mcm5
5'
3'
p1 p2
p3
p4
p5 p6
% of input
12
8
4
0
p1 p2 p3 p4 p5 p6
PCNA
3'
5'
p6
p5
p4
p3
p2 p1
% of input
20
10
0
p1 p2 p3 p4 p5 p6
B
Ssu72 wild-type cells
Pol II pausing release
Elongation
Pol II
Pol II
Promoter
CTD
P-TEFb
Ssu72
P-TEFb
Ssu72
P-TEFb
Ssu72
Fine-tuned CTD
phosphorylations
Phospho-level
pSer5
pSer7
pSer2
pThr4
Promoter
Terminator
Gene body
Ssu72-depleted cells
Elongation defect or delay
Pol II
Promoter
Recruitment
DOWN
Kinase activity
DOWN
P-TEFb
Deregulated CTD
phospho-dynamics
Phospho-level
pSer5
pSer7
pSer2
pThr4
Promoter
Terminator
Gene body

Figure S11. The Cdk9 ChIP analyses and schematic model of transcriptional regulation by mammalian Ssu72

(A) ChIP and qPCR analyses of Cdk9 against *Cdc6*, *Phyhd1*, *Mcm5* and *PCNA* genes in control and Ssu72-depleted MEFs. (B) Schematic model of transcriptional elongation activity and CTD phosphorylation profiles. See Results and Discussion sections for details.

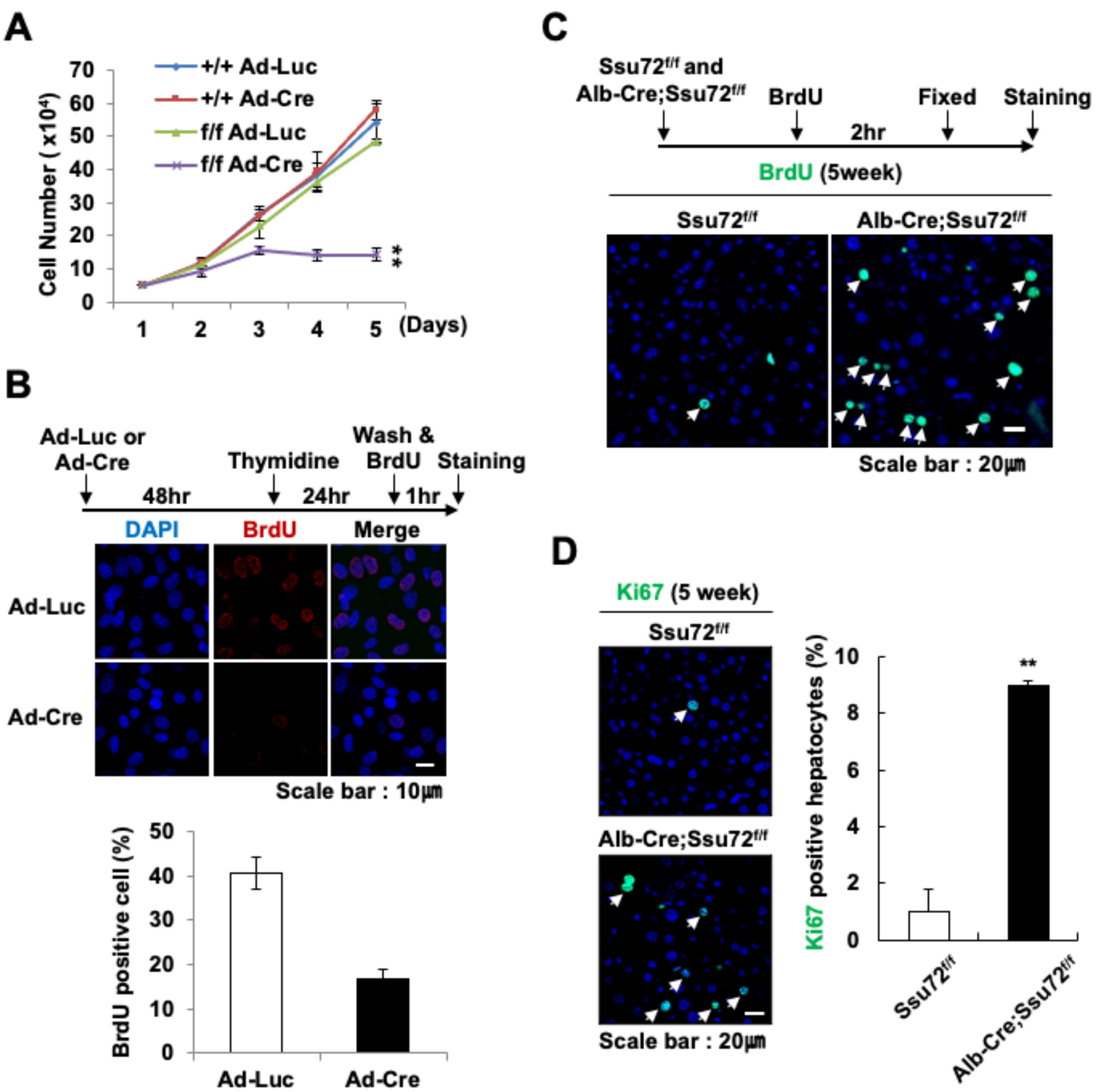

Figure S12. Ssu72 loss in mice results in distinct organ-specific phenotypes.

(A) Growth plots of Ad-Luc or Ad-Cre infected Ssu72$^{+/+}$ and Ssu72$^{f/f}$ MEFs by cell number counting. Data are presented as mean value ± SD of three independent experiments. The asterisk denotes a difference according to Student's t-test (** $p < 0.01$). (B) Ssu72$^{f/f}$ MEFs were infected with Ad-Luc or Ad-Cre, synchronized in G1/S boundary by thymidine treatment, and released for 6 h. Cells were then incorporated with BrdU for 1h. MEF cells were fixed and immunostained with anti-BrdU antibody

(upper panels). Scale bar represents 10 μm. Quantification results of BrdU-positive signals in control and Ssu72 depleted MEFs are shown in the Bottom panel. Error bars represent SD from three independent experiments. (C) BrdU was administered to 5-week-old Ssu72$^{f/f}$ and Alb-Cre; Ssu72$^{f/f}$ mice by intraperitoneal injection (IP) 2 h before surgical extracting livers. Fixed liver sections were stained with anti-BrdU antibody. Arrows indicate BrdU-positive nuclei in hepatocytes. Scale bars represent 20 μm. (D) Liver sections of 5-week-old Ssu72$^{f/f}$ and Alb-Cre; Ssu72$^{f/f}$ mice were stained with anti-Ki67 antibody (cell proliferation marker). Arrows indicate Ki67-positive nuclei in hepatocytes. Scale bars represent 20 μm.

Supplementary methods

In-gel digestion with trypsin and extraction of peptides

Protein bands from SDS-PAGE gels were excised and in-gel digested with trypsin according to established procedures [1]. In brief, protein bands were excised from stained gels and cut into pieces. The gel pieces were washed for 1 h at room temperature in 25 mM ammonium bicarbonate buffer, pH 7.8, containing 50% (v/v) acetonitrile (ACN). Following the dehydration of gel pieces in a centrifugal vacuum concentrator (Biotron, Inc., Incheon, Korea) for 10 min, gel pieces were rehydrated in 50 ng of sequencing grade trypsin solution (Promega, Madison, WI, USA). After incubation in 25 mM ammonium bicarbonate buffer, pH 7.8, at 37℃ overnight, the tryptic peptides were extracted with 100 μL of 1 % formic acid (FA) containing 50% (v/v) ACN for 20 min with mild sonication. The extracted solution was concentrated using a centrifugal vacuum concentrator. Prior to mass spectrometric analysis, the peptides solution was subjected to a desalting process using a reversed-phase column [2]. In brief, after an equilibration step with 10 ul of 5% (v/v) FA, the peptides solution was loaded on the column and washed with 10 ul of 5% (v/v) FA. The bound peptides were eluted with 8 ul of 70% ACN with 5% (v/v) formic acid.

Identification of proteins by LC-MS/MS

LC-MS/MS analysis was performed using a nanoACQUITY UPLC and LTQ-orbitrap-mass spectrometer (Thermo Electron, San Jose, CA, USA). For separation, a BEH C18 column (1.7 μm, 100 μm × 100 mm; Waters, Milford, MA, USA) was used. The mobile phase for the LC separation was 0.1% FA in deionized water (A) and 0.1% FA in ACN (B). The chromatography gradient included a linear increase from 10% B to 40% B for 21 min, from 40% B to 95% B

for 7 min, and from 90% B to 10% B for 10 min. The flow rate was 0.5 μL/min. For tandem mass spectrometry, mass spectra were acquired using data-dependent acquisition with full mass scan (300-2000 *m/z*), followed by MS/MS scans. Each MS/MS scan acquired was an average of one microscan on the LTQ. The temperature of the ion transfer tube was controlled at 275 °C, and the spray voltage was 2.0 kV. The normalized collision energy was set at 35% for MS/MS. The individual spectra from MS/MS were processed using the SEQUEST software (Thermo Quest, San Jose, CA, USA), and the generated peak lists were used to query the in-house database using the MASCOT program (Matrix Science Ltd., London, UK). We set the modifications of carbamidomethyl ('C'), deamidated ('NQ'), and oxidation ('M') for MS analysis and the tolerance of peptide mass was 10 ppm. The MS/MS ion mass tolerance was 0.8 Da, the allowance of missed cleavage was 2, and charge states (+2, +3) were taken into account for data analysis. We took only significant hits, as defined by the MASCOT probability analysis.

Two-dimensional SDS-PAGE

For the first dimension, isoelectric focusing (IEF) was performed using the IPGphor system on pH 6-11 linear gradient IPG strips (Bio-Rad). Protein (200 μg) was used for each two-dimensional gel. Samples were mixed with 200 μl of rehydration buffer [8 M urea, 2% CHAPS, 0.01% bromophenol blue, 1.2% Destreak reagent (GE Healthcare)] and 0.5% IPG buffer, and loaded in the IPGphor strip holder. The strips were then focused by the following program: rehydration for 10 h (no voltage); 0-500 V for 4 h; 500-1,000 V for 1 h; 1,000-8,000 V for 4 h; 8,000 V for 20 min; and the final phase of 500 V from 20,000-30,000 Vh. After IEF, the IPG strips were first equilibrated in equilibration buffer 1 (6 M urea, 0.5 M Tris-HCl, pH 8.8, 30% glycerol, 2% SDS, 2% 2-mercaptoethanol) for 15 min, and further in equilibration buffer 2 (6

M urea, 0.5 M Tris-HCl, pH 8.8, 30% glycerol, 2% SDS, 2.5% iodoacetamide) for 15 min. The IPG strips were transferred onto the SDS-PAGE gels and performed the immunoblotting analyses.

References

1. Bahk YY, Kim SA, Kim JS, Euh HJ, Bai GH, Cho SN, et al. Antigens secreted from *Mycobacterium tuberculosis*: identification by proteomics approach and test for diagnostic marker. Proteomics. 2004; 4: 3299–307.
2. Gobom J, Nordhoff E, Mirgorodskaya E, Ekman R, Roepstorff P. Sample purification and preparation technique based on nano-scale reversed-phase columns for the sensitive analysis of complex peptide mixtures by matrix-assisted laser desorption/ionization mass spectrometry. J Mass Spectrom. 1999; 34: 105–16.
